# Exploring Cross-Sectoral Implications of the Sustainable Development Goals: Towards a Framework for Integrating Health Equity Perspectives With the Land-Water-Energy Nexus

**DOI:** 10.3389/phrs.2022.1604362

**Published:** 2022-05-11

**Authors:** Christiana O. Onabola, Nathan Andrews, Maya K. Gislason, Henry G. Harder, Margot W. Parkes

**Affiliations:** ^1^ School of Health Sciences, University of Northern British Columbia, Prince George, BC, Canada; ^2^ Department of Global and International Studies, University of Northern British Columbia, Prince George, BC, Canada; ^3^ Faculty of Health Sciences, Simon Fraser University, Burnaby, BC, Canada

**Keywords:** health equity, cross-sectoral nexus approaches, watersheds, place-based perspectives, mapping review, land-water-energy nexus, sustainable development goals, ecosystem services pathways

## Abstract

**Objectives:** To assess existing evidence and identify gaps in the integrative framework of the Sustainable Development Goals (SDGs) for their potential to advance cross-sectoral perspectives and actions that connect health equity with the land-water-energy nexus in a watershed context.

**Methods:** Five bibliographic databases were searched from 2016 to 2021. This yielded an initial 226 publications, which were screened for titles, abstracts, and full texts on DistillerSR; resulting in a final 30 publications that were studied. These keywords defined the search terms: “health equity,” “SDGs,” “watershed,” “resource nexus,” and “cross-sectoral.”

**Results:** Thematic syntheses of debates and gaps point to the relevance of the SDGs as a cross-sectoral, integrative platform for place-based programming of the land-water-energy nexus, and to account for negative externalities and cascaded impacts on human and environmental health.

**Conclusion:** For the purpose of monitoring health equity in the contexts of interactions of land, water, and energy in rural, remote, and Indigenous contexts, and on the basis of the SDGs, this paper generates evidence to inform health equity-oriented policies, programs and practices, and to enhance health for equity-seeking populations.

## Introduction

The World Health Organization’s 2016 Shanghai Declaration on Health Promotion [1–3] stresses the imperative of leveraging interactions among the Sustainable Development Goals (SDGs)^1^ [4] to promote health and well-being. Relatedly, an overlapping body of literature [5–9] has identified the interactions at the nexus of land, water and energy as priority areas for progressing the SDGs, with far-reaching implications. [10], [6], and [7] acknowledge that challenges emerging from the land-water-energy nexus continue to impede the attainment of global sustainability goals, including health [9, 11]. Conflicts emerging from the land-water-energy nexus [12–15] not only impact on the social and ecological determinants of health [16–19], but they also lead to the inequitable distribution of risks among social groups with pre-existing vulnerabilities [20–22]. The groups most affected by resource insecurities at the interfaces of land, water, energy, and health consist of those who expend the largest share of their income to secure basic needs of water, food, and energy as necessities for health [23].

Unfortunately, inequitable impacts on health are often overlooked in land, water, energy nexus programming, particularly, in rural and Indigenous contexts [20, 24, 25]. Nexus studies of the land-water-energy domains [9, 26, 27] are beginning to highlight the need to account for disproportionate health impacts on local-settings [20, 25] already experiencing healthy inequities [20, 22]. A focus on health [12, 28, 29] has the potential to overcome some of the limitations produced through traditional siloed approaches [8, 30, 31] to managing land, water and energy systems, especially, failures to address the interconnected challenges of land, water, and energy insecurities [27], as well as the cascade of implications for human and ecosystem health [9, 16, 32, 33].

Responding to these gaps, this review explores the case for localizing the land-water-energy nexus-related SDGs in ways that resonate with the notion of “leaving no one behind”—LNOB [4]. The LNOB approach is in keeping with sub-national efforts around the world to localize the SDG agenda and develop place-relevant capacities for the local monitoring of achievements on the goals [34].

Specifically, our review explores the relevance of watersheds as an appropriate localized setting [11, 21, 35, 36] to examine nexus interlinkages of land, water, and energy systems in relation to health equity [11, 33]. Watersheds offer an integrative, ecologically coherent context to consider the land-water-energy nexus, as well as a settings-based approach [37, 38] that is consistent with the Ottawa Charter for Health Promotion [39], which emphasizes understanding health in the everyday, common-place contexts in which people live, work, learn and play [37, 38]. Watersheds also offer local representations of larger social, economic, and ecological processes and challenges [40]; as well as constitute a microcosmic unit of analysis to study the embeddedness of complex nexus challenges in sustainable development [41]. As a unit of an ecosystem, a watershed separates a larger ecosystem or landscape into interconnected geospatial units or settings, which can facilitate an inclusive mapping of environmental health inequities by supporting availability of denominator data that can unmask inequities in the social and ecological determinants of health [41, 42].

This paper responds to a growing need to connect health, equity, and place-based perspectives into land-water-energy nexus programming. It presents a narrative mapping review that aims to synthesize and visually represent available evidence and existing gaps in cross-sectoral applications of the SDGs in local nexus programming, and to explore the relevance of the SDGs as an integrative tool in this space, especially, in relation to health equity. Specific objectives are to: 1) map evidence on cross-sectoral potential of the SDGs and implications for advancing health equity within the land, water, energy nexus; 2) visualize the interlinkages of land, water and energy within the SDGs and connections to health at a watershed scale; and 3) identify knowledge gaps and integration lapses within the literature to inform the SDGs’ cross-sectional potential for health equity with focus on how indigenous knowledge and decolonizing concepts have been integrated into the nexus.

Our paper begins by describing methods used to select and analyze the literature and is followed by a presentation of results, which understands the nexus to function as an *analytical tool*, a *conceptual framework*, and a *discourse*, in ways that have the potential to foster connections across the SDGs within the watershed context. The discussion synthesizes findings associated with cross-sectoral applications of the SDGs in linking health equity with the land-water-energy nexus. The final sections provide concluding reflections and future research considerations on fostering health equity within the land-water-energy nexus through the SDGs.

## Methods

### Selection of Review Approach

Mapping reviews are increasingly used to visually depict, categorize, and synthesize measures, features and patterns of evidence existing in the broader literature with the goal of determining knowledge gaps that can inform future research [43–45]. This approach is selected to employ a broader set of questions to complement, supplement and add structure to a preliminary literature review conducted as well as to capture newly emerging literature. In addition to visual representations, a narrative synthesis is used to thematically identify existing evidence and gaps, and qualitatively explore the relationships and patterns between the findings [46, 47].

### Search Strategy

A literature search of Web of Science, MEDLINE, Science Direct, Google Scholar and Academic Search Complete was carried out with the assistance of two librarians. The date parameters were set between 2016 and 2021 to capture relevant studies published in English since September 2015, when the SDGs were launched. Keywords that made up the search terms were: “watershed,” “health equity,” “SDGs,” “resource nexus,” and “cross-sectoral.” The full list of search terms and how they were combined (using AND/OR, etc.) are presented in Supplementary Appendix A. The database search returned both peer-reviewed articles and grey literature. Grey literature predominantly arose from title searches in Google Scholar, consistent with the finding that more grey literature is found using title searches rather than full text searches in this search engine [48]. The last search was conducted on May 06, 2021. Citations of all papers identified were saved and stored in Zotero.

### Eligibility Criteria

Inclusion and exclusion criteria for the study were developed using a PICO-adapted (Problem/Perspectives-Intervention-Context/Setting-Outcome) framework [49, 50], creating a flexible guide to focus on specific criteria of interest, and is presented in Supplementary Appendix B. An article was included if it satisfied at least one of the following three conditions: 1) discusses the SDGs and cross-linkages through the lens of a nexus approach; 2) focuses on the interplay of actors, sectors or interest and distribution of power, resources and impacts in a watershed context or in the context of land/food, water, energy nexus; 3) engages with integrative, cross-sectoral, or Indigenous perspectives in a watershed context. While “health equity” was part of the keywords searched, it was not an explicit consideration in the selection criteria. This was on account of the PICO guide being used for selection criteria, where the SDGs’ integrative potential were regarded as “intervention,” and cross-sectoral possibilities taken for “outcome.” In this regard, health equity was considered as one of the potential “outcomes” of cross-sectoral possibilities arising at the land-water-energy nexus. Moreover, since previous studies already stressed that the land-water-energy nexus does not often account for health and equity concerns, we did not want to miss relevant articles that did not discuss health equity in explicit terms. Rather, literature that addressed health equity considerations were identified throughout the screening stages of the review.

### Screening, Quality Appraisal and Data Extraction

The initial selection of articles retrieved from the database searches were passed through title, abstract and full-text screening on DistillerSR [51]. A list of the included and excluded studies can be found in Supplementary Appendices C, D respectively. To be included in the final subset of articles that met the inclusion criteria, the grey literature was appraised using the AACODS checklist [52], which is a standard quality appraisal tool that assesses on authority, accuracy, coverage, objectivity, date and significance (Supplementary Appendix E). After ascertaining the quality of included studies, key data for each study were extracted, using a PICO-adapted framework [49, 50] in Microsoft Excel, to collate categories of information, which were later distilled into relevant themes for discussion.

### Data Analysis

Thematic analysis [47, 53] was used to identify themes, and capture patterns and trends with a focus on creating snapshot profiles of land, water and energy interlinkages in the SDGs, in relation to categorized themes, existing evidence and identified gaps.

Consistent with the review objectives, the first focus of the analyses was to identify trends and patterns by dividing the included studies into three nexus role categories, as proposed by [54] and expanded by [55]. Analyzing the three nexus roles (as an analytical tool, as a conceptual framework and as a discourse) helped bring together theoretical, conceptual, and value-laden approaches within the nexus integration agenda. Specific attention was paid to cross-sectoral activity given its relevance for health equity integration into the land-water-energy nexus in that it fosters an understanding of the cross-sectoral capacities of the goals and their targets and potential opportunities that exist for promoting health equity when linking the land-water-energy nexus and SDGs indicators [30]. The second analyses focused on mapping the interlinkages of land, water and energy within the SDGs and their connections to health at a watershed scale. The third thematically identified knowledge gaps and integration lapses to inform thinking about the SDGs’ cross-sectional potential for linking health equity with the land-water-energy nexus in small, rural, and Indigenous contexts.

## Results

Our findings are separated into four sections starting with the results of the database search in bibliographic databases. This is followed by summary tables of the categorization of nexus roles describing how these roles are characterized in the literature and pathways of connections in relation to cross-sectoral potential of the SDG’s and implications for health equity. Next, we depict a visual map of interlinkages of land, water and energy within the SDGs and implications for health at a watershed scale. These results further lead to a visual representation of high priority nexus gaps, underscoring integration lapses for health equity in rural, remote indigenous contexts.

### Results of Database Search

**FIGURE 1:**
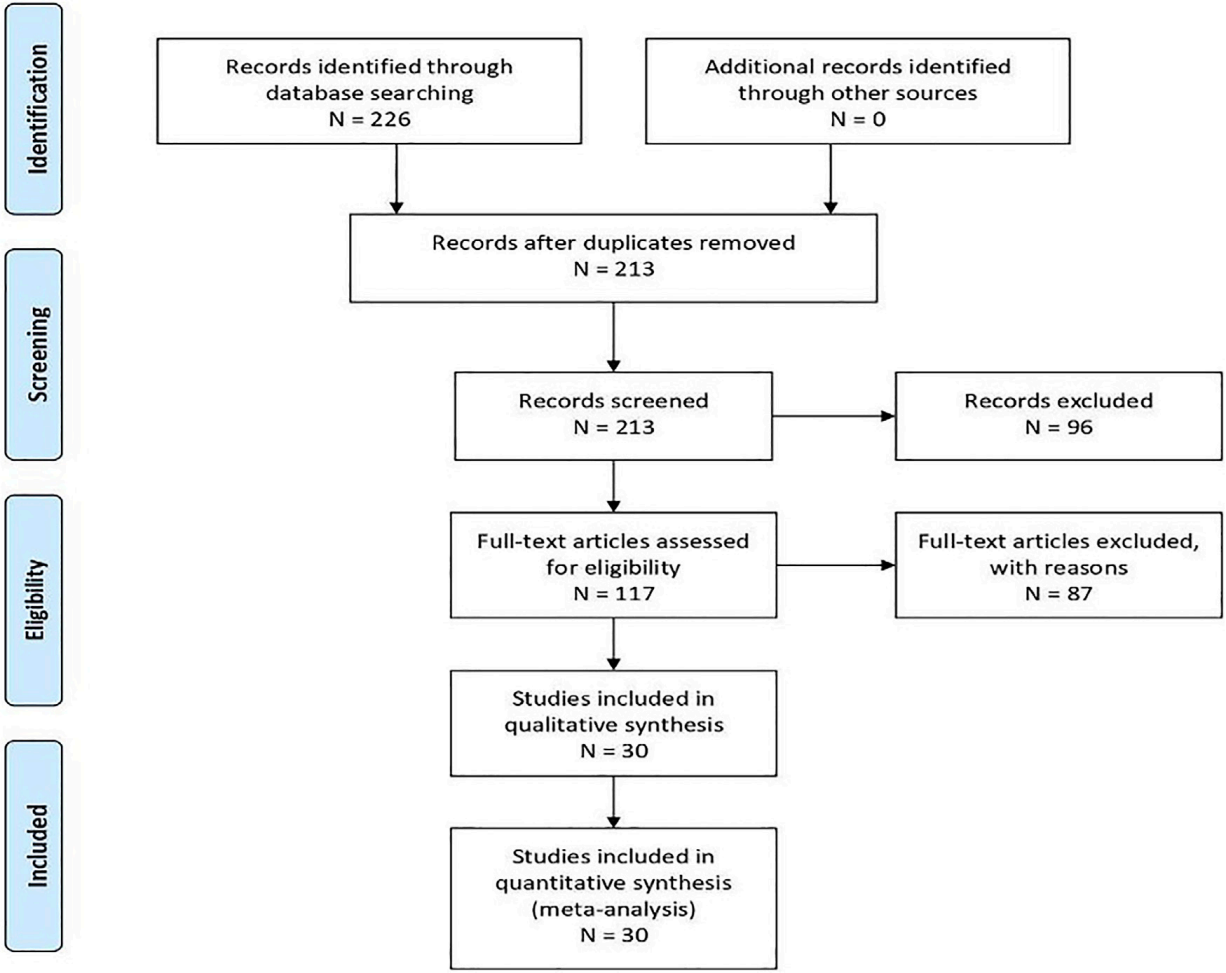
Prisma Flow Chart: A flow chart representing the study selection and screening steps Creative by DistillerSR (c) (Prince George, Canada, 2021).

Of the 226 returned articles from the database search, 13 duplicates were removed, and 213 were screened. Level 1 (title and abstract) screening excluded 96 studies, and 117 were moved to level 2 (full texts) screening, which resulted in 30 articles being included in the study. All five grey literature items were included following AACODS quality appraisal checklist [52]. Figure 1 depicts the study selection and screening steps in a flowchart.

### Summary of Included Studies Categorized by Nexus Roles

Albrecht et al [55], the most highly cited article in our sample, presents a systematic review of existing nexus approaches and proposes that operationalizing nexus thinking around land, water and energy resources can have three roles: 1) a*s an analytical tool,* 2) *as a conceptual framework*, and 3) *as discourse.* As an *analytical too*l, nexus approaches employ quantitative or qualitative methods, or a combination of both, to study interlinkages among water, energy, and land systems. As a *conceptual framework*, the nexus approach draws on interlinkages between land, water, and energy to advance policy coherence. As *discourse*, the nexus concept is used to frame inherent challenges in fostering cross-sectoral collaboration [55].

Table 1 below provides an overview of the key characteristics of the 30 studies arranged by publication dates, describing nexus roles (as an *analytical tool*, a *conceptual framework*, a *discourse*) across the 30 studies, as well as relationships with the SDGs and links to social determinants of health and health equity. These tables are structured to distinguish journal articles from grey literature and appreciate differences in publication trends between the two. Table 1 presents the range of approaches to the nexus role among the 30 included studies. Without exhibiting ties with other roles, ten focused on analytical approaches, four on conceptual frameworks, and seven employed a discourse. The remaining eight employed two roles, and one study connected across the three roles. From the sample, there appears to be an overall trend, over the past 5 years, towards the use of analytical approaches to understanding interlinkages of complex systems as well as the use of discourses to frame challenges in cross-sectoral collaboration within and across the nexus.

**TABLE 1:** Key characteristics of identified Journal articles and Grey Literature (Prince George, Canada, 2021).

Author (Year) Title (Journal Articles)	Role of Nexus framing: see Notes (1,2,3)	Description: Key connections with Land-Water-Energy nexus and the SDGs, and examples of the pathways and relationships connecting the SDGs to the Social determinants of Health and Equity.
[56] The five-node resource nexus at sea	1	Explored the potential for developing conservation and policy interventions to preserve threatened ecosystem functions and services in the marine water–energy–biomass–minerals–land nexus through conservation of ecologically critical natural habitats that sustain these services - which determine the health and wellbeing of humans and ecosystems [66]
[83] Energy, water, and food: towards a critical nexus approach	3	Developed frames for the challenges within the water-energy-food nexus and articulated the conceptual, methodological, and practical solutions for advancing cross-sectoral integration [83]
[73] Beyond zero sum game allocations: expanding resources potentials through reduced interdependencies and increased resource nexus synergies	2	Explored how to deal with the interdependencies of water, energy, and food systems through use of improved policies, technologies, and adapted human behaviors; such that foster system resilience and cross-sectoral communication [73]
[54] The Water-Energy-Food Nexus: A systematic review of methods for nexus assessment	3	Use of frames to articulate methodological limitations of nexus analytical tools in achieving cross-connections for health and equity [54]
[61] Advancing the implementation of SDGs in Brazil by integrating water-energy nexus and legal principles for better governance	1,2	Explored interlinkages of water and energy nexus and drew on an understanding of interconnections to reveal challenges to integration within the SDGs and to policy coherence in nexus outcomes [61]
[29] Nexus approaches to global sustainable development	2,3	In the use of nexus approaches for uncovering synergies and detecting trade-offs, it is crucial for the nexus to internalize accounting for and reconciling spill-over effects and cascaded impacts on human and environmental health externalized from trade-off interactions [29]
[58] Assessing the State of the Water-Energy-Food (WEF) Nexus in South Africa	1	Employed the platform of an established link between WEF indicators and SDGs indicators to examine pathways between the water, energy, food (WEF) nexus and rural livelihoods, health, and well-being in southern Africa [58]
[65] How extractive industries affect health: Political economy underpinnings and pathways	1, 3	Employed system thinking perspectives and frames to draw attention to pathways by which extractive industries affect health outcomes and engender health inequities [65]
[75] Water–energy–food nexus: a platform for implementing the Sustainable Development Goals	1, 2, 3	Examined tight interconnections within and across water, energy, and food systems. Proposed that SDG criteria should be the baseline and minimum development goals to be pursued in implementation of the water-energy- land nexus at any scale [75]
[69] Opportunities and Trade-offs among BECCS and the Food, Water, Energy, Biodiversity, and Social Systems Nexus at Regional Scales.	2	Developed a conceptual framework that incorporated biodiversity and social systems as part of the water- energy-food nexus. The framework was used as an interdisciplinary platform to analyze the trade-offs and opportunities among emerging policy strategies at a river basin scale [69]
[57] The Water–Food–Energy Nexus: Power, Politics, and Justice	3	Framed the nexus challenges to advancing cross-sectoral integration. This combined perspectives and concerns on the politics of the nexus, power sharing, equity, and justice [57]
[79] Complexity versus simplicity in water energy food nexus (WEF) assessment tools	1	Recognizing unique constraints and complexities across “resource hotspots,” the authors developed a tool consisting of a simple-complex spectrum for assessing complexity and appropriation of nexus tools [79]
[58] The water–energy–food nexus as a tool to transform rural livelihoods and well-being in southern Africa	1, 2	An analytical tool was armed with capabilities to interrogate complex systems for livelihood and health impacts of the resource nexus. The tool was later used as a conceptual framework to support decision making for coherent policies [72]
[82] Structuring an integrated water-energy-food nexus assessment of a local wind energy desalination system for irrigation	1	Used a novel analytical approach for integrated assessment of water, energy, and food systems in a local desalination case study in the Canary Islands, Spain [82]
[77] From a few security indices to the FEW Security Index: Consistency in global food, energy, and water security assessment	1, 3	Analyzed the methodological inconsistencies associated with various indices used in nexus approaches and discussed underlying assumptions to identify and explain these inconsistencies [77]
[78] Linking Environmental Policy Integration and the Water-Energy-Land-(Food-)Nexus: A Review of the European Union’s Energy, Water, and Agricultural Policies.	2	Used the nexus as one of the conceptual frameworks to evaluate European energy, water, and agricultural policies; and the extent to which integration was inculcated into the design and implementation of these policies [78]
[64] Sustainable development as the ultimate target of adopting a nexus approach to resources management	1	Stressed the necessity of making nexus approaches more robust with innovative tools that will factor in ecosystem services pathways and make for comprehensive unravelling of interlinkages and cross-sectoral externalities—important for propelling resources management towards achieving Sustainable development [64]
[76] Toward understanding the convergence of researcher and stakeholder perspectives related to water-energy-food (WEF) challenges: The case of San Antonio, Texas	2, 3	Evaluated levels of convergence in perspectives and challenges of cross-sectoral communication between water, energy, and food stakeholders and researchers [76]
[26] Local community perceptions toward livelihood and water–energy–food nexus: A perspective on food security.	3	Examined the framing of nexus contributions to livelihoods in a local community. This was important to identify missing links on how nexus resources can enhance living conditions [26]
[68] Sustaining the ecological functions of the Litani River Basin, Lebanon.	1	Examined water quality and quantity indicators using Sustainable Development Goal 6 (SDG 6) to provide a guide on water availability and sustainable management of water and sanitation for all [68]
[67] Linking reservoir ecosystems research to the sustainable development goals	1	Examined interlinkages between reservoir ecosystems (wetlands, dams, etc) and the SDGs. 71% of the SDGs have established synergies with these ecosystems. This accentuates the significance of ecosystem services to health and sustainable development [67]
[56] A critical analysis of the food-energy-water nexus in the Kootenai River Basin	3	Framed the nexus to advance integration of social and environmental dimensions at a river basin scale [56]
[60] A Nexus Approach to Water, Energy, and Food (WEF) Security in Northern Canada	1	Analyzed interlinkages of synergies and trade-offs between WEF-nexus related SDGs of Goal 2, 6 and 7. A higher extent of synergies than trade-offs between the targets revealed the interdependence of water, energy and food insecurity challenges and opportunities for exploring synergistic effects of targets within each domain to address these challenges [60]
[80] Gateway to the perspectives of the Food-Energy-Water nexus	3	Explored five key perspectives used to frame the nexus and the motivations for use of the perspectives, which are: Ecosystems, waste management, institutional change, trust, and learning process perspectives [80]
[59] The potential of water security in leveraging Agenda 2030	1	Statistical examination of interlinkages among the SDGs to find interconnections and correlations [59]

Author (Year) Title (Grey Literature)	Role of Nexus framing: see Notes (1,2,3)	Description: key connections with Land-Water-Energy nexus and SDGs, and examples of the pathways and relationships connecting SDGs to the social determinants of health and equity.
[81] The UN, global governance, and the SDGs	2, 3	Presents the SDGs as a governance tool and a sustainability instrument for cross-sectoral integration across issues, sectors, scales, and regions within and across the water-energy-food nexus [81]
[25] Development of water-energy-food nexus conceptual framework for Bangladesh	2	Employed an understanding of WEF nexus interlinkages as a basis to influence policy outcomes in Bangladesh [25]
[74] Governing the Water-Energy-Food Nexus ISAP2018 Approach for Creating Synergies and Managing Trade-offs	1,2	Examined interlinkages between SDG 2, SDG 6, SDG 7, and their targets, and drew on interlinked challenges to make recommendations for coherence in policy structures [74]
[63] Forests, Forest People, and UN 2030 Agenda’s Ethical Mandate: “LEAVE NO ONE BEHIND”	1	Focused on interlinkages of forest, trees outside forests, and agroforestry (FTA) sectors with each of the SDGs and contributions to sustainable livelihoods and development. This was examined in the context of isolated Indigenous Peoples and local communities (IPLCs) and aligns with the Leave No One Behind pledge [63]
[71] The development of the Water-Energy-Food (WEF) Nexus Index and its application to the Southern African Development Community.	3	Entailed conceptualization OF WEF-nexus gaps and challenges to cross-sectoral integration including nexus neglect of distributional justice [71]

Land-Water-Energy nexus role, relationships with the Sustainable Development Goals, and links to social determinants of health and health equity; ordered by publication date (oldest to newest).

Notes: Role of Nexus Framing—(1) As an Analytical Tool: employs quantitative or qualitative methods to study nexus interactions. (2) As a Conceptual Framework: draws on interlinkages between land, water, and energy to advance policy coherence. (3) As a Discourse: frames inherent challenges in fostering cross-sectoral collaboration.

### Mapping Evidence on Cross-Sectoral Potential of the Sustainable Development Goals and Implications for Advancing Health Equity

The nexus term and the SDGs have both been used to connote principles and processes of integration [8, 31]. They both possess elements of an integrated human-environment framework [56] and serve multiple and wide-ranging objectives that link one to the other. Table 2 below cross-links the three identified roles of nexus framing to inform the evidence gathered on potential cross-sectoral applications of the SDGs in fostering sensitivity to health equity in the land-water-energy nexus.

**TABLE 2:** Characterizing Nexus-informed Cross-sectoral potential of the Sustainable Development Goals and Implications for Advancing Health Equity (Prince George, Canada, 2021).

Nexus-informed Cross-sectoral Potential of the Sustainable Development Goals (SDGs)	Current Evidence for Advancing Health Equity
The SDGs as an A*nalytical tool*	Uses a systems-thinking basis to unpack interlinkages and draw correlations with the social determinants of health [59, 60, 73]
The SDGs as a *Conceptual Framework*	A paradigm to navigate coordination challenges in analyzing the nature and extent of trade-offs which breed injustices and inequities in socio-environmental outcomes [58, 61, 71, 72]
The SDGs as a *Discourse*	Uses value-laden judgements and frames to draw attention to governance, power sharing and equity concerns in terms of these lines of questioning: “*Integration for whom? Who leads the coordinated efforts? Whose interests are integrated? Whose are traded-off?*” [26, 57,65, 66, 81]

Considering that the “nexus” term has been used interchangeably with the SDGs in recent literature [31], there is a burgeoning “vice versa” opportunity for using nexus variables to facilitate the localization of the SDGs and adapt SDGs target to local realities and priorities [30, 57]. Table 2 provides a summary of SDGs’ cross-sectoral roles cross-linked with nexus framings. The table also profiles examples of the pathways and relationships connecting the SDGs to the social determinants of health and equity. In relation to the SDGs’ role as *an analytical tool* and usage in the included literature, the SDGs framework is used to unpack interlinkages and identify options for maximizing synergies and balancing trade-offs [30]. This is often facilitated by linking nexus variables with SDG indicators and mapping areas of indicator overlap or correlations with the social and ecological determinants of health [30].

Mabhaudhi et al. [58] employed the water-energy-food nexus as an analytical tool and linked nexus indices with SDGs’ indicators, in a systems-thinking manner, to explore impacts on rural livelihoods, health, and well-being in Southern Africa. [59] and [60] statistically assessed interlinkages among nexus-related SDGs 2, 6 and 7 to uncover deep interconnections of resource insecurities and opportunities for unlocking synergies and balancing trade-offs. In [30] and [60], application of the nexus, in an analytical role to the SDGs, entailed collapsing nexus variables to overlap with SDGs indicators in a way that drew correlations with the social determinants of health and furthered the SDGs.

As *a conceptual framework*, the SDGs can be promoted to resolve challenges often associated with the coordination of knowledge, interests, perspectives, and factors, and to address co-production failures in analyzing the nature and extent of trade-offs, which often produce injustices and inequities in socio-environmental outcomes. [61] and [27] employed the SDGs as a normative, conceptual framework for sustainable development, drawing on an appreciation of the complex relationships between water and energy, and multiple cross-cutting targets that cater to more than one goal. As a conceptual framework, the SDGs are promoted to resolve inherent challenges often associated with interlinkages, tending to stem from inadequate considerations of the potentials for synergies, and failures to analyze the nature and extent of trade-offs. The SDGs, as a conceptual framework, can bring into perspective prospective challenges and coordinate mechanisms needed for coherent nexus solutions, which include navigating different management approaches and tackling bottlenecks which also reinscribe injustices and inequities in socio-environmental outcomes [61]). In the light of the power relations and coordination challenges associated with the transdisciplinary character of cross-sectoral nexus framing, the SDGs *as a discourse* [62] uses value-laden judgements to draw attention to governance, power sharing, distributive justice and equity concerns around access to and use of resources as well as the burden of resource use impacts [57].

### Mapping Interlinkages of Land, Water and Energy Within the Sustainable Development Goals and Connections to Health at a Watershed Scale

**FIGURE 2:**
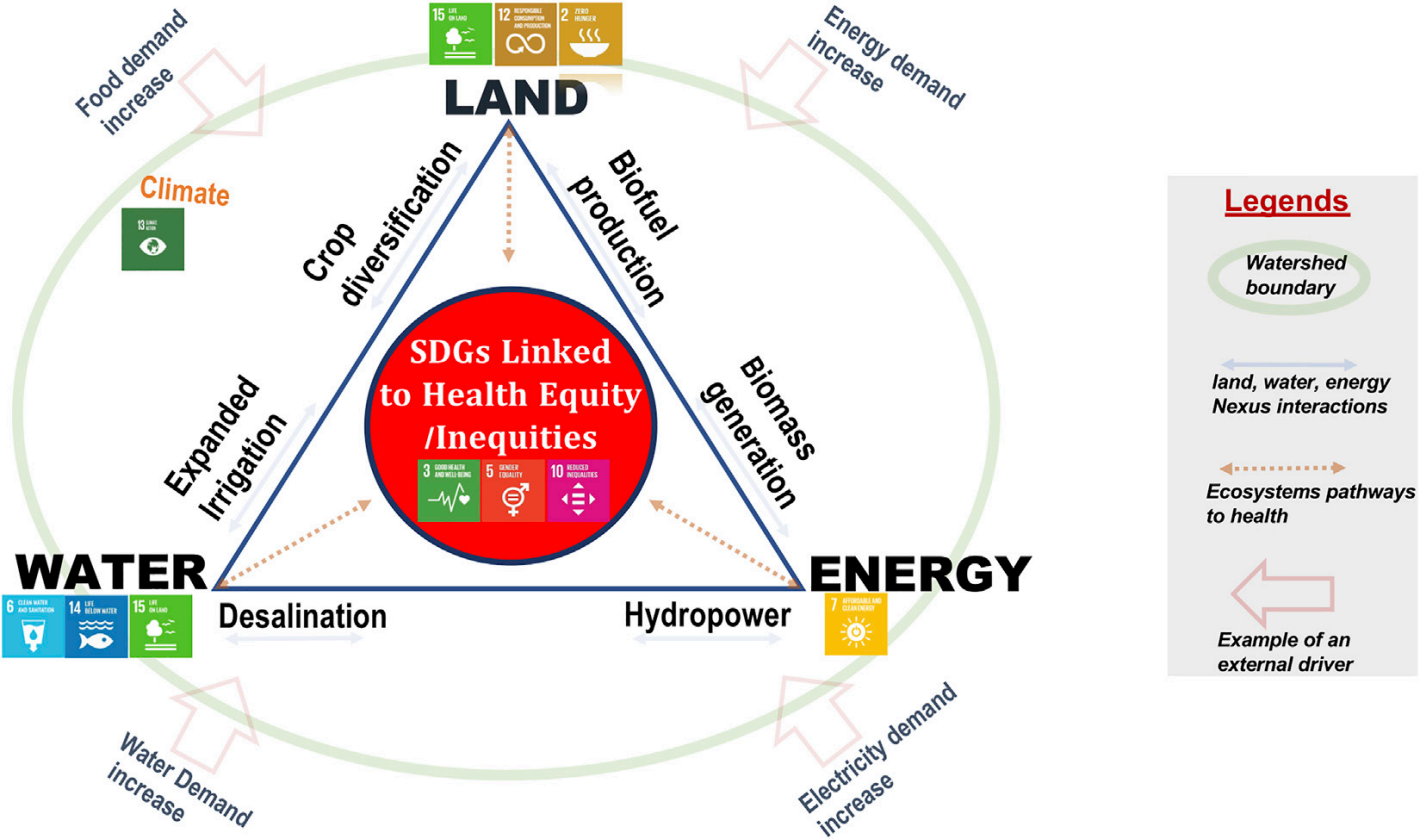
Resource dynamics of land, water and energy at a watershed scale (Prince George, Canada, 2021).

**FIGURE 3:**
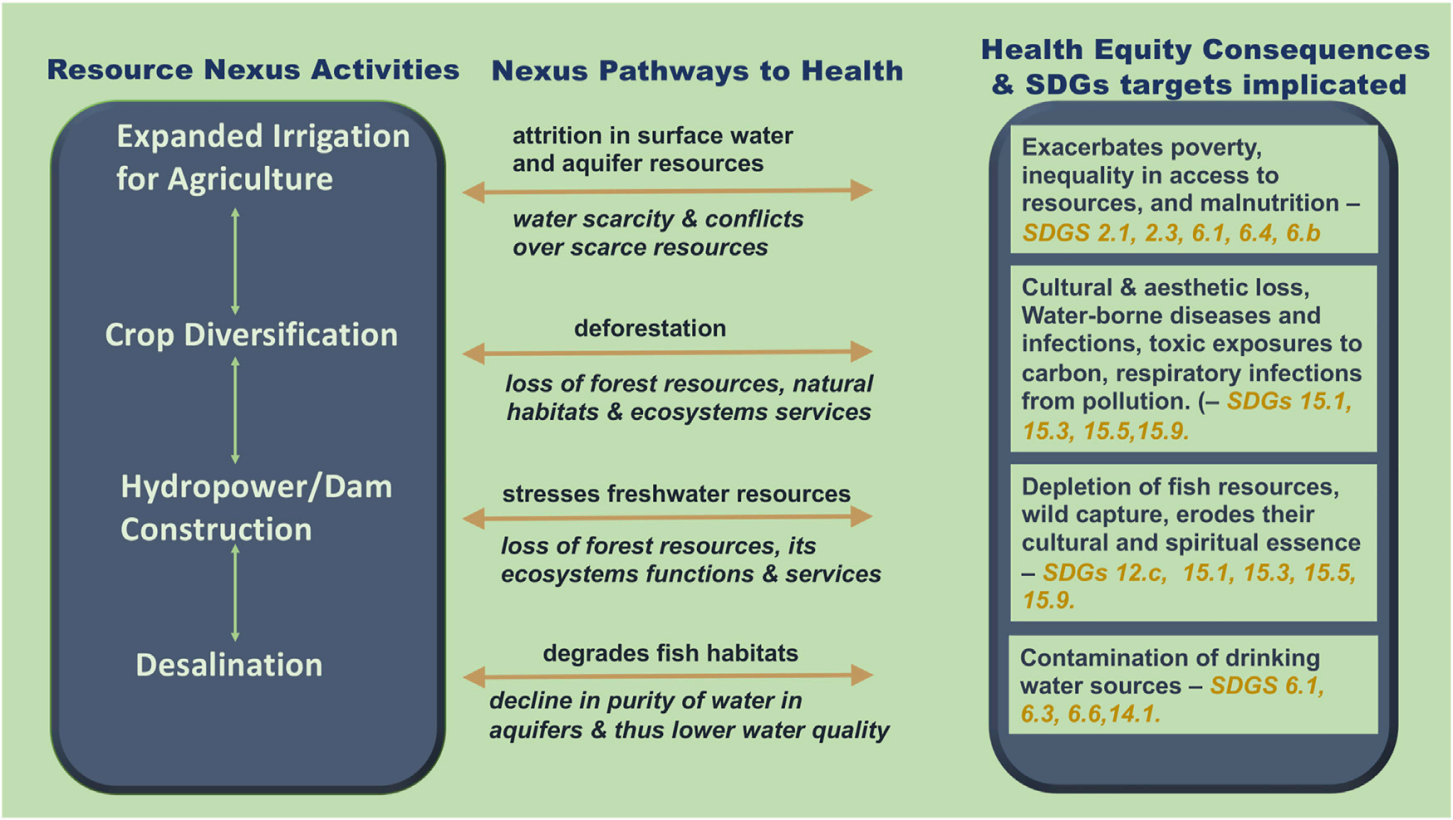
Schematic diagram of interactions among land, water and energy systems, pathways to health equity impacts and corresponding implicated targets within the Sustainable Development Goals’ framework (Prince George, Canada, 2021).

In Figures 2, 3 below, the SDGs are ascribed with goals and targets for each of land, water, and energy resources, and Figure 2 depicts that land (SDGs 2, 15 and 12), water (SDGs 6, 14 and 15), and energy (SDG 7) are closely interlinked. The contextual boundary is a watershed, which is considered a resource nexus hotspot where interaction dynamics around the use of land, water and energy resources for production and consumption are very tangible. In a watershed context, numerous factors are implicated in driving the dynamics (Figure 2) around the use of land, water and energy resources for production and consumption [63]. These drivers, shown in Figure 2, can be as direct as increased demands for food, water, and energy or as indirect as climate change, increasing human population, urbanization, globalization and human civilization. The responses to these drivers are encapsulated in the resource dynamics of production and consumption, which consists of activities of dam construction and hydropower generation, coal mining, crop diversification, expanded irrigation, biofuel production, biomass generation and desalination.

Figure 3 below depicts potential pathways [64] by which resource nexus projects can impinge on the health of humans [65] biodiversity [66], and natural ecosystems [67] as well as impact on determinants of health [60, 68] through disrupting the ecosystem services of provisioning, regulating, supporting, and preserving culture [64, 69].

Desalination, for instance, is an energy demanding process that removes salt from sea water to make it potable for drinking. The process is, however, complicated by leeching of the chemicals used into the soil, thus contaminating water storage in aquifers, thereby lowering water quality [70]. There is also a possibility of brine dumping that contaminates the food chain for both marine life and human consumption. These contamination chains will impact food and water security and cause disparities in access to safe drinking water, sanitation services, and affordable, nutritious food. The pollution chains reveal how nexus activities often follow ecosystem pathways in how they influence the social determinants of health. These connections between the resource nexus and the ecosystem service pathways which influence health and wellbeing have not been factored into nexus assessments [64].

### Mapping Nexus Gaps and Lapses in Relation to the Sustainable Development Goals: Implications for Advancing Health Equity

**FIGURE 4:**
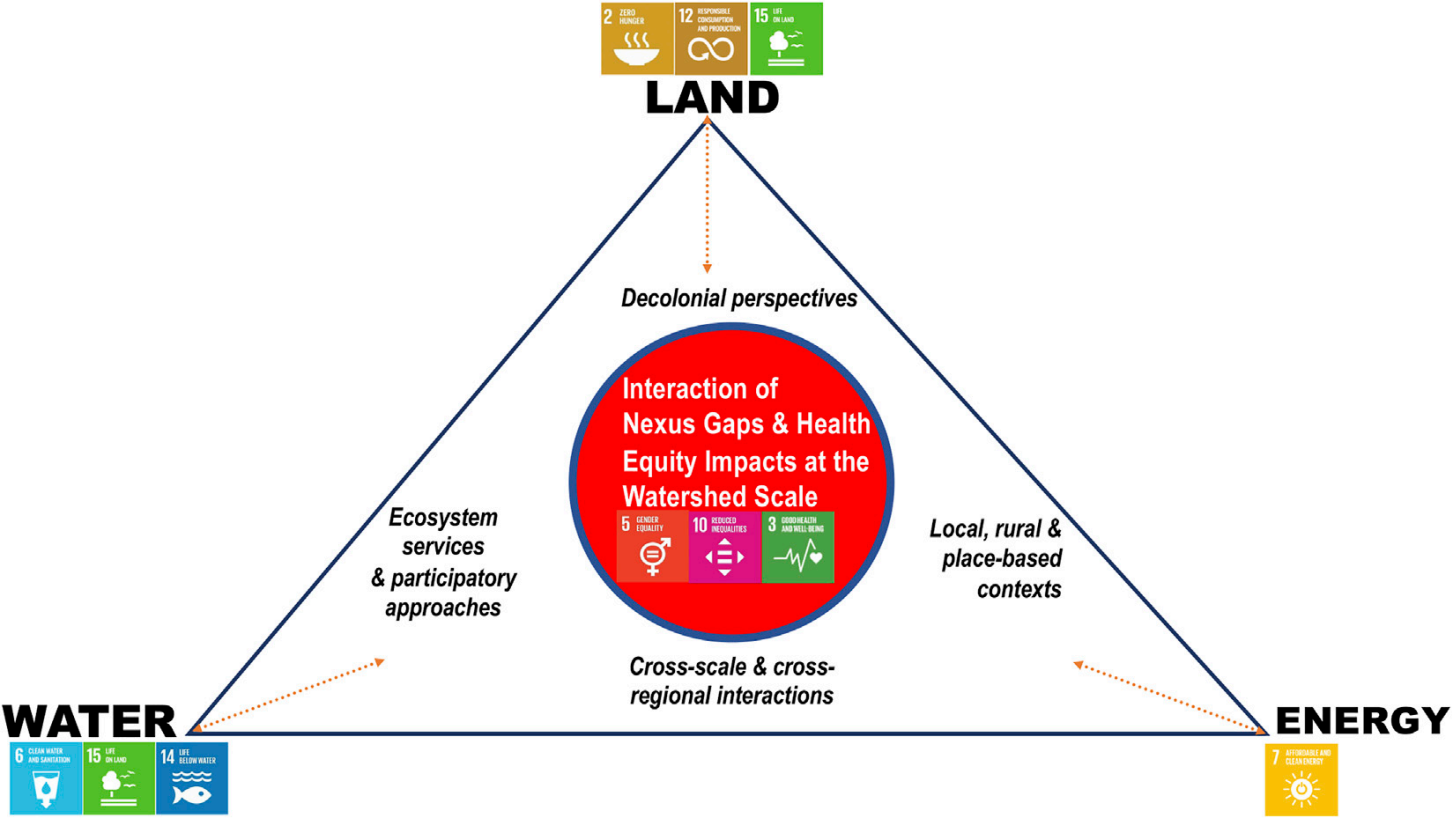
A framework for addressing knowledge gaps and cross-sectoral health equity impacts of the Land, Water, Energy nexus at the Watershed Scale (Prince George, Canada, 2021).

The results presented here are based on thematic grouping of knowledge gaps and integration lapses within nexus literature and explain how the SDGs’ framework in its cross-sectoral strengths accounts for the nexus gaps. Figure 4 is a visual map of knowledge gaps within the land, water-energy nexus and it represents a proposed simple framework of often overlooked dynamics within the nexus to which attention should be accorded for addressing health equity concerns in rural, remote, and Indigenous contexts.

Resource insecurities disproportionately impact rural and remote communities and impinge on Indigenous Peoples’ self-determining goals, socio-environmental values that connect land to health, and ways of knowing and being. In relation to the gaps grouped under decolonial perspectives and place-based contexts in Figure 4, our analysis has highlighted ways in which literature focused on nexus approaches have not tended to be inclusive of theoretical perspectives grounded in decolonial scholarship [57, 60, 63], local and place-based approaches, and Indigenizing ideas [30, 57, 60, 63, 64]. Addressing these gaps and engaging meaningfully with these literatures could enhance options for the SDG’s to provide a basis from which to encourage effective cross-sectoral engagement, bridge epistemological divides and address the strengths and limitations of different knowledge domains and approaches [61, 64].

Another integration lapse within the nexus is related to failures to promote participatory approaches. Nexus tools are largely quantitative; thus, there is a need to consider qualitative, participatory approaches [30, 55], which highlight socio-political nuances, such as power, politics, equity, distributional justice, identities, and emotions in nexus framing and conceptualization of challenges and solutions [55, 57, 61, 71]. Moreover, as a result of a missing focus on social and environmental dimensions of sustainability within the nexus, there are significant gaps in understanding ecosystem service pathways of land, water and energy interactions that underlie health inequities [30, 55, 57, 64]. However, the SDGs, which offer a way to address human-nature coupled systems, also offer the integrative capacity to redress these missing sustainability dimensions.

Additionally, there are scalar lapses associated with the nexus in connection with failures to account for health and equity externalities from cross-scale and cross-regional interactions of land, water, and energy systems [30]. Current nexus tools often focus on a specific place or context [30, 55]. This precludes considerations for cross-sectoral and cross regional interactions that often result in leakages or spillover effects. Attending to cross-scalar issues will support the potential of the SDG’s to provide a meta-coupling framework [30] that facilitates the integration of human-nature interactions (people and ecosystems) across spatial scales and builds on concepts such as globalization to address multi-scalar, socio-ecological challenges occurring between adjacent or distant systems at local, regional, and global scales.

## Discussion

This discussion considers findings from the mapping review in relation to the challenges of localising and understanding health equity dynamics of the SDG’s as well as recommendations for future studies investigating possible pathways to center health equity in the land, water, energy nexus at a watershed scale.

Our review identified numerous ways that water, energy, and land insecurities drive disparities in the determinants of health for human wellbeing and ecosystems [58, 60, 66–68, 72], particularly, in rural, remote, and Indigenous contexts where the use and extraction of natural resources [57] create downstream health inequities. For example, Natcher and Ingram report incidences of higher rates of water, energy, and food (WEF) insecurity in rural and remote communities in Northern Canada, where residents struggle with wide disparities in access to safe drinking water, sanitation services, as well as affordable energy and nutritious food [60].

Figure 2 depicts examples of land-water-energy interactions unfolding within watersheds, which serve as resource nexus hotspots [73], as multi-sectoral and multi-stakeholder spaces [57, 64] and as sites of interdependent land, water and energy resource insecurities [57, 64]. Issues of resource insecurities associated with the nexus of land, water, and energy illustrate why it is impossible to consider one dimension without taking into consideration the others. One example is derived from the Kootenai River basin, a transboundary river bordering some parts of British Columbia in Canada and the United States [57]. Complex systems linkages of land, water and energy resources take shape through intensive resource development activities such as agricultural expansion, dam construction and hydropower generation and open pit coal mining. Land, water, and energy are inputs into these resource activities, and efforts to address challenges in one sector impacts as well as produces emergent issues in other sectors [60, 73, 74]. Hence, nexus actors are compelled to make an increasing number of trade-off decisions that produce health externalities and present communities with conflicts that impact health outcomes. A few studies [27, 57, 58, 60, 63, 72] traced these health conflicts to resource insecurity challenges with concerns about disproportionate environmental burdens and the uneven distribution of health risks and environmental exposures in social groups (humans and biodiversity) with pre-existing vulnerabilities. Some studies [57, 60, 69, 75] employed an environmental justice lens to analyze socio-environmental conflicts emanating from trade-off interactions among land, water, energy resources and explored gendered and intersectional implications of resource use among social groups. These analyses dovetail with social, economic, and environmental determinants of health and health equity at a watershed scale and raise questions about the use of these resources in terms of: “Who has access to the resources? For what purposes? At what cost? With what impacts? And who bears the burden of the impacts? [75].”

In line with the socio-environmental lapses discussed above, and depicted in Figures 2, 3, many of the studies [30, 55, 76–84] pointed to a lack of attention within existing nexus methods to systematically unpacking synergies and trade-offs between social and environmental issues across a range of contexts and scales. Our findings (Figure 4), characterize these as scalar and theoretical lapses and link them to a lack of attention to decolonial perspectives that are needed to address impacts on those rural, remote, and Indigenous communities most affected by resource development activities. We underscore that the sustainability framework of the SDG agenda can contribute to the nexus agenda by addressing sustainability dimensions emerging between social and environmental domains through engaging principles of integration such as the notions of intergenerational equity, environmental protection, and the linking of the economic, social, cultural, and environmental dimensions of sustainability.

When considering the cross-sectoral relevance of the SDG’s, our review identifies delays and barriers to engagement within rural and Indigenous contexts which also further entrench continued epistemological divides between western and Indigenous conceptualizations of sustainability and development [34, 84]. A pertinent question is whether (and how) the SDGs can help to bridge the two ideologies and inculcate Indigenous Peoples’ self-determining goals, socio-environmental values, ways of knowing and being into nexus issues. Findings presented in *Mapping Evidence on Cross-Sectoral Potential of the Sustainable Development Goals and Implications for Advancing Health Equity* section, illustrate ways that the SDGs have the potential to offer an integrative socio-ecological framework that can accommodate the strengths and limitations of different knowledge domains, approaches, and perspectives, while also offering a cross-sectoral platform that can foster communication and co-production among diverse actors and interest groups [76].

Finally, studying the links between the land-water-energy nexus and SDGs indicators underscores the importance of future studies exploring the cross-sectoral impacts on health equity [58, 75]. Connecting SDG indicators relating to land, water and energy, and making clear links to health indicators at a watershed scale are areas for future work that highlight the potential value of an SDGs-data-driven approach within watersheds that centres health equity in this nexus. These future studies will also need to address data-related challenges at a watershed scale [41, 42]. The land-water-energy-health equity nexus has the potential to be strengthened through increased emphasis on ecosystems services and related pathways [64] by which the nexus of land, water and energy have impacts on livelihoods, human well-being and the other species that depend on these services [66, 67, 69, 81]. This emphasis may also enhance the emergence of community-based application of the SDGs focused on this nexus [27, 57, 58, 60, 68, 72, 75], with the potential to inform decisions around land, water, and energy insecurities as well as how these underlie the social and ecological determinants of health inequities. The land, water, energy nexus can also be applied to analyze specific resource issues such as forestry [63], hydropower [67], and desalination [83], with an emphasis on understanding impacts on health outcomes, such as the distribution of the impacts of socio-ecological determinants of health. If future research is to be effective in supporting greater equity and sustainability within nexus systems of land, water, and energy [66, 75], the scale and geography of the nexus will be important to consider along with the socio-political nuances and particular socio-ecological dimensions contouring these settings [57, 84].

### Strengths and Limitations of the Study

The findings from this review need to be considered with reference to both limitations and strengths. One limitation is the use of only studies published in English and between 2016 and 2021. Relevant studies published in other languages and before 2016, just after the SDGs were launched in September of 2015, might have been missed. The inclusion of grey literature created a mixed pool of study types and can be considered as both a limitation and a strength. The inclusion of grey literature provides important contextual information on equity lapses in the nexus literature and on the socio-economic and socio-ecological implications for local communities and Indigenous populations. Pertaining to the interests of this research in how indigenous contexts have been advanced into land-water-energy nexus programming, the term—“Indigeneity”—was employed as a keyword along with other keywords introduced in the search strategy to explore how nexus approaches have integrated Indigenous ways of knowing and being. “Indigeneity"—as a keyword—was broken into search terms that included a range of possible synonyms in the literature: (indigen* OR decoloniz* OR aboriginal OR “* ecological knowledge” OR “first nations” OR metis OR Inuit OR “native people”). However, combining these “Indigeneity” search terms with the search terms of other keywords used in the search strategy, using “AND”, returned “0” for most of the database searches, except for Google scholar, which returned a few relevant articles. One interpretation of this outcome is that this indicates limited research conducted within the nexus domain that considers the integration of Indigenous socio-environmental outcomes and self-determination goals. To ensure uniformity of keywords considered across the search databases, “Indigeneity” and synonyms were explicitly removed from the search terms but considered as an implicit inclusion criterion.

### Conclusion

There is a wealth of overlapping literature [30, 61, 63, 64, 66, 74, 83] on how prioritizing a focus on the nexus of land, water and energy systems can accelerate progress in meeting the SDGs. The water-energy-land nexus offers a potential tool for centering health across scales and contexts, in keeping with the WHO’s Shanghai’s Declaration on leveraging interactions to promote health in the SDGs [1, 3]. However, several existing nexus approaches do not actively foster cross-sectoral integration [78, 79], system thinking, transdisciplinarity [30, 75, 84], nor place-based considerations in support of promoting the equitable integration of multiple perspectives, knowledges, and needs within decision-making and policy outcomes [77].

This review has addressed an important knowledge gap by mapping interlinkages of land, water, energy, and health equity within the SDGs at a watershed scale. It has characterized the cross-sectoral potential of the SDGs to advance health equity within the land, water, energy nexus, and illustrated how integration lapses may compromise the capacity of the land-water-energy nexus to address health equity considerations in rural, remote, and Indigenous contexts. Our review [73] also identifies the SDGS as a promising driver for progressing the nexus integration agenda to foster sensitivity to health equity within the nexus of land, water and energy. It focuses on the cross-sectoral potential of the SDGs to foster an appreciation of the impacts of the land water, energy nexus on health outcomes, such as the distribution of the socio-ecological determinants of health. For the purpose of monitoring health equity in the context of interactions of land, water and energy systems, this paper generates evidence to inform health equity-oriented policies, programs, and practices, and to enhance health for equity-seeking populations. Our work identifies further research needs to address knowledge gaps regarding health equity and the SDGs at the scale of watersheds, including closer attention to unmet needs of equity-seeking populations, priorities for Indigenous communities, and a closer focus on health equity as an integral dynamic within the land-water-energy nexus.

## Footnotes

1 The United Nations 2030 Sustainable Development Goals (SDGs) is a blueprint of 17 goals, 169 targets and 231 indicators (4) framed by member states to tackle interconnected sustainability challenges across economic, social, and environmental domains.
