# Supplementary material for: Exploring Cross-Sectoral Implications of the Sustainable Development Goals: Towards a Framework for Integrating Health Equity Perspectives With the Land-Water-Energy Nexus
Source: Public Health Rev. 2022 May 11;43:1604362. doi: 10.3389/phrs.2022.1604362 (PMC9131490; doi:10.3389/phrs.2022.1604362)
Supplement: Supplementary file 1 [file Table1.DOCX]

Agardy, T. (2017). The five-node resource nexus at sea. In *Routledge Handbook of the Resource Nexus* (pp. 406–423). Routledge.

Albrecht, T. R., Crootof, A., & Scott, C. A. (2018). The Water-Energy-Food Nexus: A systematic review of methods for nexus assessment. *Geoscientific Model Development Discussions*, *13*(4), 1–33. a9h.

Allouche, J., Middleton, C., & Gyawali, D. (2019). *The Water–Food–Energy Nexus: Power, Politics, and Justice*. Routledge.

Biswas, P. P. (2018). *Development of water-energy-food nexus conceptual framework for Bangladesh*.

Carvalho, P., & Spataru, C. (2018). Advancing the implementation of SDGs in Brazil by integrating water-energy nexus and legal principles for better governance. *Sustainability in Environment*, *3*(3), 277–304.

Caucci, S., Zhang, L., Locher-Krause, K., & Hülsmann, S. (2020). 6 Sustainable development as the ultimate target of adopting a nexus approach to resource s managemen t. *Sustainable Development and Resource Productivity*, 67.

Daher, B., Hannibal, B., Mohtar, R. H., & Portney, K. (2020). Toward understanding the convergence of researcher and stakeholder perspectives related to water-energy-food (WEF) challenges: The case of San Antonio, Texas. *Environmental Science & Policy*, *104*, 20–35. <https://doi.org/10.1016/j.envsci.2019.10.020>

Dargin, J., Daher, B., & Mohtar, R. H. (2019). Complexity versus simplicity in water energy food nexus (WEF) assessment tools. *Science of The Total Environment*, *650*, 1566–1575. <https://doi.org/10.1016/j.scitotenv.2018.09.080>

Darwish, T., Shaban, A., Masih, I., Jaafar, H., Jomaa, I., & Simaika, J. P. (2021). Sustaining the ecological functions of the Litani River Basin, Lebanon. *International Journal of River Basin Management*, 1–15. a9h.

Gregersen, H., El-Lakany, H., & Frechette, A. (n.d.). *Forests, Forest People, and UN 2030 Agenda’s Ethical Mandate:“LEAVE NO ONE BEHIND.”*

Guo, Z., Boeing, W. J., Borgomeo, E., Xu, Y., & Weng, Y. (2021). Linking reservoir ecosystems research to the sustainable development goals. *Science of The Total Environment*, 146769.

Ivanova, M., & Escobar-Pemberthy, N. (2017). The UN, global governance and the SDGs. In *Routledge Handbook of the Resource Nexus* (pp. 486–502). Routledge.

Leck, H., Fitzpatrick, D., & Burchell, K. (2017). Energy, water and food: Towards a critical nexus approach. In *Handbook on the Geographies of Energy*. Edward Elgar Publishing.

Liu, J., Hull, V., Godfray, H. C. J., Tilman, D., Gleick, P., Hoff, H., Pahl-Wostl, C., Xu, Z., Chung, M. G., & Sun, J. (2018). Nexus approaches to global sustainable development. *Nature Sustainability*, *1*(9), 466–476.

Loodin, N., Keith, T., Arynova, A., Rosenblum, Z., & Ruzmatova, Z. (2021). A critical analysis of the food-energy-water nexus in the Kootenai River Basin. *SUSTAINABLE WATER RESOURCES MANAGEMENT*, *7*(3). <https://doi.org/10.1007/s40899-021-00508-w>

Mabhaudhi, T, Simpson, G., Badenhorst, J., Mohammed, M., Motongera, T., Senzanje, A., & Jewitt, A. (2018). Assessing the State of the Water-Energy-Food (WEF) Nexus in South Africa. *South Africa: Water Research Commission & University of KwaZulu-Natal.(WRC Report No KV 365/18) Date of Access*, *1*, 0365–18.

Mabhaudhi, Tafadzwanashe, Nhamo, L., Mpandeli, S., Nhemachena, C., Senzanje, A., Sobratee, N., Chivenge, P. P., Slotow, R., Naidoo, D., & Liphadzi, S. (2019). The water–energy–food nexus as a tool to transform rural livelihoods and well-being in southern Africa. *International Journal of Environmental Research and Public Health*, *16*(16), 2970.

Mitra, B. K., Pham, N.-B., Amanuma, N., & Yoshida, T. (n.d.). Governing a Water-Energy-Food Nexus Approach: Creating Synergies and. *Governance for Integrated Solutions to Sustainable Development and Climate Change*, 93.

Mohtar, R. H., & Daher, B. (2017). Beyond zero sum game allocations: Expanding resources potentials through reduced interdependencies and increased resource nexus synergies. *Biotechnology and Bioprocess Engineering / Process Systems Engineering*, *18*, 84–89. <https://doi.org/10.1016/j.coche.2017.09.002>

Natcher, D., & Ingram, S. (2021). A Nexus Approach to Water, Energy, and Food Security in Northern Canada. *ARCTIC*, *74*(1), 1–11.

Proctor, K., Tabatabaie, S. M. H., & Murthy, G. S. (2021). Gateway to the perspectives of the Food-Energy-Water nexus. *Science of The Total Environment*, *764*, 142852. <https://doi.org/10.1016/j.scitotenv.2020.142852>

Schrecker, T., Birn, A.-E., & Aguilera, M. (2018). How extractive industries affect health: Political economy underpinnings and pathways. *Health & Place*, *52*, 135–147. <https://doi.org/10.1016/j.healthplace.2018.05.005>

Serrano-Tovar, T., Peñate Suárez, B., Musicki, A., e la Fuente Bencomo, J. A., Cabello, V., & Giampietro, M. (2019). Structuring an integrated water-energy-food nexus assessment of a local wind energy desalination system for irrigation. *Science of The Total Environment*, *689*, 945–957. <https://doi.org/10.1016/j.scitotenv.2019.06.422>

Simpson, G. B. (2020). *The development of the Water-Energy-Food Nexus Index and its application to the Southern African Development Community.*

Stephan, R. M., Mohtar, R. H., Daher, B., Embid Irujo, A., Hillers, A., Ganter, J. C., Karlberg, L., Martin, L., Nairizi, S., & Rodriguez, D. J. (2018). Water–energy–food nexus: A platform for implementing the Sustainable Development Goals. *Water International*, *43*(3), 472–479.

Stoy, P. C., Ahmed, S., Jarchow, M., Rashford, B., Swanson, D., Albeke, S., Bromley, G., Brookshire, E. N. J., Dixon, M. D., & Haggerty, J. (2018). Opportunities and Trade-offs among BECCS and the Food, Water, Energy, Biodiversity, and Social Systems Nexus at Regional Scales. *BioScience*, *68*(2), 100–111. a9h.

Taka, M., Ahopelto, L., Fallon, A., Heino, M., Kallio, M., Kinnunen, P., Niva, V., & Varis, O. (2021). The potential of water security in leveraging Agenda 2030. *One Earth*, *4*(2), 258–268.

Venghaus, S., & Dieken, S. (2019). From a few security indices to the FEW Security Index: Consistency in global food, energy and water security assessment. *Sustainable Production and Consumption*, *20*, 342–355. <https://doi.org/10.1016/j.spc.2019.08.002>

Venghaus, S., Märker, C., Dieken, S., & Siekmann, F. (2019). Linking environmental policy integration and the water-energy-land-(food-) nexus: A review of the European Union’s energy, water, and agricultural policies. *Energies*, *12*(23), 4446.

Wolde, Z., Wei, W., Kunpeng, W., & Ketema, H. (2020). Local community perceptions toward livelihood and water–energy–food nexus: A perspective on food security. *Food & Energy Security*, *9*(3), 1–15. a9h.
