# Supplementary material for: Exploring Cross-Sectoral Implications of the Sustainable Development Goals: Towards a Framework for Integrating Health Equity Perspectives With the Land-Water-Energy Nexus
Source: Public Health Rev. 2022 May 11;43:1604362. doi: 10.3389/phrs.2022.1604362 (PMC9131490; doi:10.3389/phrs.2022.1604362)
Supplement: Supplementary file 2 [file Table2.DOCX]

Al-Saidi, M., & Elagib, N. A. (2017). Towards understanding the integrative approach of the water, energy and food nexus. *Science of the Total Environment*, *574*, 1131–1139.

Altamirano, M., van Bodegom, A., van der Linden, N., Rijke, H. de, Verhagen, J., Bucx, T., Boccalon, A., & van der Zwaan, B. (2018). *Operationalizing the WEF nexus: Quantifying the trade-offs and synergies between the water, energy and food sectors*.

Artioli, F., Acuto, M., & McArthur, J. (2017). The water-energy-food nexus: An integration agenda and implications for urban governance. *Political Geography*, *61*, 215–223.

Belda Gonzalez, A. (2018). *The Water-Energy-Agriculture nexus in Jordan: A case study on As-Samra wastewater treatment plant in the LowerJordan River Basin*.

Benites-Lazaro, L., Giatti, L., Junior, W. S., & Giarolla, A. (2020). Land-water-food nexus of biofuels: Discourse and policy debates in Brazil. *Environmental Development*, *33*, 100491.

Bidoglio, G., Vanham, D., Bouraoui, F., & Barchiesi, S. (2018). The Water-Energy-Food-Ecosystems (WEFE) Nexus. In *Reference Module in Earth Systems and Environmental Sciences*. Elsevier.

Bijl, D. L., Bogaart, P. W., Dekker, S. C., & van Vuuren, D. P. (2018). Unpacking the nexus: Different spatial scales for water, food and energy. *Global Environmental Change*, *48*, 22–31. <https://doi.org/10.1016/j.gloenvcha.2017.11.005>

Bird, J., Prathapar, S., Perry, C., Mohtar, R., & Tollefson, L. (2020). *ENABLING POLICY ENVIRONMENT FOR WATER, FOOD AND ENERGY SECURITY*.

Bluemling, B., Tai, H.-S., & Choe, H. (2021). Boundaries, limits, landscapes and flows: An analytical framework for boundaries in natural resource management. *Journal of Environmental Management*, *285*, 112129. <https://doi.org/10.1016/j.jenvman.2021.112129>

Botai, J., Botai, C., Ncongwane, K., Mpandeli, S., Nhamo, L., Masinde, M., Adeola, A., Mengistu, M., Tazvinga, H., & Murambadoro, M. (2021). *A Review of the Water–Energy–Food Nexus Research in Africa. Sustainability 2021, 13, 1762*.

Botai, J. O., Botai, C. M., Ncongwane, K. P., Mpandeli, S., Nhamo, L., Masinde, M., Adeola, A. M., Mengistu, M. G., Tazvinga, H., & Murambadoro, M. D. (2021). A Review of the Water–Energy–Food Nexus Research in Africa. *Sustainability*, *13*(4), 1762.

Braun, A. C. (2021). Encroached by pine and eucalyptus? A grounded theory on an environmental conflict between forest industry and smallholder livelihoods in Chile. *Journal of Rural Studies*, *82*, 107–120. <https://doi.org/10.1016/j.jrurstud.2021.01.029>

Buechler, S., Vázquez-García, V., Martínez-Molina, K. G., & Sosa-Capistrán, D. M. (2020). Patriarchy and (electric) power? A feminist political ecology of solar energy use in Mexico and the United States. *Energy Research & Social Science*, *70*, 101743. <https://doi.org/10.1016/j.erss.2020.101743>

Cabello, V., Renner, A., & Giampietro, M. (2019). Relational analysis of the resource nexus in arid land crop production. *Advances in Water Resources*, *130*, 258–269. <https://doi.org/10.1016/j.advwatres.2019.06.014>

Caiado Couto, L., Campos, L. C., Fonseca-Zang, W., Zang, J., & Bleischwitz, R. (2021). Water, waste, energy and food nexus in Brazil: Identifying a resource interlinkage research agenda through a systematic review. *Renewable and Sustainable Energy Reviews*, *138*, 110554. <https://doi.org/10.1016/j.rser.2020.110554>

Carmona-Moreno, C., Dondeynaz, C., & Biedler, M. (2019). *Position Paper on Water, Energy, Food and Ecosystems (WEFE) Nexus and Sustainable Development Goals (SDGs).* Publications Office of the European Union.

Casazza, M., Xue, J., Du, S., Liu, G., & Ulgiati, S. (2021). Simulations of scenarios for urban household water and energy consumption. *PLoS ONE*, *16*(4), 1–15. a9h.

Chang, N.-B., Hossain, U., Valencia, A., Qiu, J., Zheng, Q. P., Gu, L., Chen, M., Lu, J.-W., Pires, A., & Kaandorp, C. (2020). Integrative technology hubs for urban food-energy-water nexuses and cost-benefit-risk tradeoffs (II): Design strategies for urban sustainability. *Critical Reviews in Environmental Science and Technology*, 1–51.

Chen, C.-F., Feng, K.-L., & Ma, H. (2020). Uncover the interdependent environmental impacts associated with the water-energy-food nexus under resource management strategies. *Resources, Conservation and Recycling*, *160*, 104909. <https://doi.org/10.1016/j.resconrec.2020.104909>

Chen, I.-C., Wang, Y.-H., Lin, W., & Ma, H. (2020). Assessing the risk of the food-energy-water nexus of urban metabolism: A case study of Kinmen Island, Taiwan. *Ecological Indicators*, *110*, 105861. <https://doi.org/10.1016/j.ecolind.2019.105861>

Chen, L.-H., Li, P.-C., Lin, Y., Chen, I.-C., Ma, H., & Yu, C.-P. (2021). Establishing a quantification process for nexus repercussions to mitigate environmental impacts in a water-energy interdependency network. *Resources, Conservation and Recycling*, *171*, 105628. <https://doi.org/10.1016/j.resconrec.2021.105628>

Crane, M., Lloyd, S., Haines, A., Ding, D., Hutchinson, E., Belesova, K., Davies, M., Osrin, D., Zimmermann, N., Capon, A., Wilkinson, P., & Turcu, C. (2021). Transforming cities for sustainability: A health perspective. *Environment International*, *147*, 106366. <https://doi.org/10.1016/j.envint.2020.106366>

Cremades, R., Mitter, H., Tudose, N. C., Sanchez-Plaza, A., Graves, A., Broekman, A., Bender, S., Giupponi, C., Koundouri, P., & Bahri, M. (2019). Ten principles to integrate the water-energy-land nexus with climate services for co-producing local and regional integrated assessments. *Science of the Total Environment*, *693*, 133662.

Daher, B., Mohtar, R. H., Pistikopoulos, E. N., Portney, K. E., Kaiser, R., & Saad, W. (2018). Developing socio-techno-economic-political (STEP) solutions for addressing resource nexus hotspots. *Sustainability*, *10*(2), 512.

Dawoud, M. A. H. (2017). Water, energy, and food security nexus in the west asian region. *Water-Energy-Food Nexus: Principles and Practices, Eds P. Abdul Salam, S. Shrestha, V. Prasad Pandey, and AK Anal (Washington, DC: John Wiley and Sons, Inc.)*, 163–180.

Dogaru, D., Mauser, W., Balteanu, D., Krimly, T., Lippert, C., Sima, M., Szolgay, J., Kohnova, S., Hanel, M., & Nikolova, M. (2019). Irrigation Water Use in the Danube Basin: Facts, Governance and Approach to Sustainability. *Journal of Environmental Geography*, *12*(3–4), 1–12.

e Macedo, M. B., Gomes, M. N., e Oliveira, T. R. P., Giacomoni, M. H., Imani, M., Zhang, K. F., o Lago, C. A. F., & Mendiondo, E. M. (n.d.). Low Impact Development practices in the context of United Nations Sustainable Development Goals: A new concept, lessons learned and challenges. *Critical Reviews in Environmental Science and Technology*. <https://doi.org/10.1080/10643389.2021.1886889>

Endo, A., Yamada, M., Miyashita, Y., Sugimoto, R., Ishii, A., Nishijima, J., Fujii, M., Kato, T., Hamamoto, H., Kimura, M., Kumazawa, T., & Qi, J. (2020). Dynamics of water–energy–food nexus methodology, methods, and tools. *Environmental Monitoring Assessment: Water-Energy-Food Nexus*, *13*, 46–60. <https://doi.org/10.1016/j.coesh.2019.10.004>

Gao, T., Fang, D., & Chen, B. (2020). Multi-regional input-output and linkage analysis for water-PM2.5 nexus. *Applied Energy*, *268*, 115018. <https://doi.org/10.1016/j.apenergy.2020.115018>

Golubchikov, O. (2020). People-Smart Sustainable Cities. *Available at SSRN 3757563*.

Haberl, H., Schmid, M., Haas, W., Wiedenhofer, D., Rau, H., & Winiwarter, V. (2021). Stocks, flows, services and practices: Nexus approaches to sustainable social metabolism. *Ecological Economics*, *182*, 106949. <https://doi.org/10.1016/j.ecolecon.2021.106949>

Hezri, A. (2018). *An Overview Study of Water-Energy-Food Nexus in Malaysia*.

Hoffmann, H. K., Sander, K., Brüntrup, M., & Sieber, S. (2017). Applying the Water-Energy-Food Nexus to the Charcoal Value Chain. *Frontiers in Environmental Science*, *5*, 84.

Hogeboom, R., Borsje, B., Deribe, M., van der Meer, F., Mehvar, S., Meyer, M., Özerol, G., Hoekstra, A., & Nelson, A. (2021). Resilience Meets the Water–Energy–Food Nexus: Mapping the Research Landscape. Front. *Environ. Sci*, *9*, 630395.

Hogeboom, R. J., Borsje, B. W., Deribe, M. M., Van Der Meer, F. D., Mehvar, S., Meyer, M. A., Özerol, G., Hoekstra, A. Y., & Nelson, A. D. (2021). Resilience Meets the Water–Energy–Food Nexus: Mapping the Research Landscape. *Frontiers in Environmental Science*, *9*, 38.

Howarth, C., & Monasterolo, I. (2016). Understanding barriers to decision making in the UK energy-food-water nexus: The added value of interdisciplinary approaches. *Environmental Science & Policy*, *61*, 53–60. <https://doi.org/10.1016/j.envsci.2016.03.014>

Hülsmann, S., & Jampani, M. (2021). The Nexus Approach as a Tool for Resources Management in Resilient Cities and Multifunctional Land-Use Systems. In *A Nexus Approach for Sustainable Development* (Vol. 717, pp. 1–13). Springer. <https://doi.org/10.1016/j.scitotenv.2020.137264>

Laspidou, C. S., Mellios, N. K., Spyropoulou, A. E., Kofinas, D. T., & Papadopoulou, M. P. (2020). Systems thinking on the resource nexus: Modeling and visualisation tools to identify critical interlinkages for resilient and sustainable societies and institutions. *Science of the Total Environment*, *717*, 137264.

Lázaro, L. L. B., Giatti, L. L., & e Oliveira, J. A. P. (2021). Water-energy-food nexus approach at the core of businesses–How businesses in the bioenergy sector in Brazil are responding to integrated challenges? *Journal of Cleaner Production*, 127102.

Lehmann, S. (2018). Implementing the Urban Nexus approach for improved resource-efficiency of developing cities in Southeast-Asia. *City, Culture and Society*, *13*, 46–56. <https://doi.org/10.1016/j.ccs.2017.10.003>

Liu, J., Yang, H., Cudennec, C., Gain, A. K., Hoff, H., Lawford, R., Qi, J., e Strasser, L., Yillia, P. T., & Zheng, C. (2020). Challenges in operationalizing the water-energy-food nexus (Reprinted from HYDROLOGICAL SCIENCES JOURNAL, vol 62, pg 1714-1720, 2017). *Hydrological Sciences Journal-Journal Des Sciences Hydrologiques*, *65*, 1714–1720. <https://doi.org/10.1080/02626667.2017.1353695>

Liu, J., Yang, H., Cudennec, C., Gain, A. K., Hoff, H., Lawford, R., Qi, J., Strasser, L. de, Yillia, P. T., & Zheng, C. (2017). Challenges in operationalizing the water–energy–food nexus. *Hydrological Sciences Journal/Journal Des Sciences Hydrologiques*, *62*(11), 1714–1720. a9h.

Liu, Junguo, Yang, H., Cudennec, C., Gain, A. K., Hoff, H., Lawford, R., Qi, J., Strasser, L. de, Yillia, P., & Zheng, C. (2017). Challenges in operationalizing the water–energy–food nexus. *Hydrological Sciences Journal*, *62*(11), 1714–1720.

Lv, H., Yang, L., Zhou, J., Zhang, X., Wu, W., Li, Y., & Jiang, D. (2020). Water resource synergy management in response to climate change in China: From the perspective of urban metabolism. *Resources, Conservation and Recycling*, *163*, 105095.

McGrane, S. J., Acuto, M., Artioli, F., Chen, P., Yu, C., Robert, C., Julian, F., Wharton, G., Green, N., Helfgott, A., Larcom, S., McCann, J. A., O’Reilly, P., Salmoral, G., Scott, M., Todman, L. C., Gevelt, T., & Yan, X. (2019). Scaling the nexus: Towards integrated frameworks for analysing water, energy and food. *Geographical Journal*, *185*(4), 419–431. a9h.

Mguni, P., van Vliet, B., Spaargaren, G., Nakirya, D., Osuret, J., Isunju, J. B., Ssekamatte, T., & Mugambe, R. (2020). What could go wrong with cooking? Exploring vulnerability at the water, energy and food Nexus in Kampala through a social practices lens. *Global Environmental Change*, *63*, 102086. <https://doi.org/10.1016/j.gloenvcha.2020.102086>

Mohtar, R. H., & Daher, B. (2019). Lessons learned: Creating an interdisciplinary team and using a nexus approach to address a resource hotspot. *Science of The Total Environment*, *650*, 105–110. <https://doi.org/10.1016/j.scitotenv.2018.08.406>

Momblanch, A., Papadimitriou, L., Jain, S. K., Kulkarni, A., Ojha, C. S. P., Adeloye, A. J., & Holman, I. P. (2019). Untangling the water-food-energy-environment nexus for global change adaptation in a complex Himalayan water resource system. *Science of the Total Environment*, *655*, 35–47. <https://doi.org/10.1016/j.scitotenv.2018.11.045>

Mroue, A. M., Mohtar, R. H., Pistikopoulos, E. N., & Holtzapple, M. T. (2019). Energy Portfolio Assessment Tool (EPAT): Sustainable energy planning using the WEF nexus approach – Texas case. *Science of The Total Environment*, *648*, 1649–1664. <https://doi.org/10.1016/j.scitotenv.2018.08.135>

Müller-Mahn, D., & Gebreyes, M. (2019). Controversial connections: The water-energy-food nexus in the Blue Nile basin of Ethiopia. *Land*, *8*(9), 135.

Munoz Castillo, R., Feng, K., Sun, L., Guilhoto, J., Pfister, S., Miralles-Wilhelm, F., & Hubacek, K. (2019). The land-water nexus of biofuel production in Brazil: Analysis of synergies and trade-offs using a multiregional input-output model. *Journal of Cleaner Production*, *214*, 52–61. <https://doi.org/10.1016/j.jclepro.2018.12.264>

Nawab, A., Liu, G., Meng, F., Hao, Y., Zhang, Y., Hu, Y., & Casazza, M. (2019). Exploring urban energy-water nexus embodied in domestic and international trade: A case of Shanghai. *Journal of Cleaner Production*, *223*, 522–535. <https://doi.org/10.1016/j.jclepro.2019.03.119>

Nhamo, L., Ndlela, B., Mpandeli, S., & Mabhaudhi, T. (2020). The Water-Energy-Food Nexus as an Adaptation Strategy for Achieving Sustainable Livelihoods at a Local Level. *Sustainability*, *12*(20). <https://doi.org/10.3390/su12208582>

Nhamo, L., Ndlela, B., Nhemachena, C., Mabhaudhi, T., Mpandeli, S., & Matchaya, G. (2018). The Water-Energy-Food Nexus: Climate Risks and Opportunities in Southern Africa. *Water*, *10*(5). <https://doi.org/10.3390/w10050567>

Nhamo, Luxon, Mpandeli, S., Senzanje, A., Liphadzi, S., Naidoo, D., Modi, A. T., & Mabhaudhi, T. (2021). Transitioning Toward Sustainable Development Through the Water–Energy–Food Nexus. In *Sustaining Tomorrow via Innovative Engineering* (pp. 311–332). World Scientific.

Olawuyi, D. (2020). Sustainable development and the water-energy-food nexus: Legal challenges and emerging solutions. *Environmental Science & Policy*, *103*, 1–9.

Opejin, A. K., Aggarwal, R. M., White, D. D., Jones, J. L., Maciejewski, R., Mascaro, G., & Sarjoughian, H. S. (2020). A bibliometric analysis of food-energy-water nexus literature. *Sustainability*, *12*(3), 1112.

Pahl-Wostl, C., Gorris, P., Jager, N., Koch, L., Lebel, L., Stein, C., Venghaus, S., & Withanachchi, S. (2021). Scale-related governance challenges in the water–energy–food nexus: Toward a diagnostic approach. *Sustainability Science*, *16*(2), 615–629.

Pandey, V. P., & Shrestha, S. (2017). Evolution of the nexus as a policy and development discourse. *Water-Energy-Food Nexus: Principles and Practices*, *1*, 11–20.

Purwanto, A., Sušnik, J., Suryadi, F. X., & Fraiture, C. de. (2021). Water-energy-food nexus: Critical review, practical applications, and prospects for future research. *Sustainability*, *13*(4), 1919.

Ramos, E. P., Howells, M., Sridharan, V., Engström, R. E., Taliotis, C., Mentis, D., Gardumi, F., e Strasser, L., Pappis, I., & Balderrama, G. P. (2021). The climate, land, energy, and water systems (CLEWs) framework: A retrospective of activities and advances to 2019. *Environmental Research Letters*, *16*(3), 033003.

Rasul, G. (2016). Managing the food, water, and energy nexus for achieving the Sustainable Development Goals in South Asia. *Environmental Development*, *18*, 14–25.

Rasul, G., Neupane, N., Pasakhala, B., Gurung, P., Nepal, A. K., & Sharma, E. (2020). Deepening regional cooperation for enhancing water, energy, and food security and addressing COVID-19 challenges in the Hindu Kush Himalayan Region. In *Water Resources and Economic Processes* (pp. 139–164). Routledge.

Rasul, G., & Sharma, B. (2016). The nexus approach to water–energy–food security: An option for adaptation to climate change. *Climate Policy*, *16*(6), 682–702.

Santos Da Silva, S. R., Miralles-Wilhelm, F., Muñoz-Castillo, R., Clarke, L. E., Braun, C. J., Delgado, A., Edmonds, J. A., Hejazi, M., Horing, J., Horowitz, R., Kyle, P., Link, R., Patel, P., Turner, S., & McJeon, H. C. (2019). The Paris pledges and the energy-water-land nexus in Latin America: Exploring implications of greenhouse gas emission reductions. *PLoS ONE*, *14*(4), 1–26. a9h.

Schlör, H., Märker, C., & Venghaus, S. (2021). Developing a nexus systems thinking test –A qualitative multi- and mixed methods analysis. *Renewable and Sustainable Energy Reviews*, *138*, 110543. <https://doi.org/10.1016/j.rser.2020.110543>

Sharma, E. (2020). Golam Rasul, Nilhari Neupane, Binaya Pasakhala, Prakriti Gurung, Apsara Karki Nepal, and. *Water Resources and Economic Processes*, 139.

Spataru, C. (2017). The five-node resource nexus dynamics. *Routledge Handbook of the Resource Nexus*.

Stein, C., Pahl-Wostl, C., & Barron, J. (2018). Towards a relational understanding of the water-energy-food nexus: An analysis of embeddedness and governance in the Upper Blue Nile region of Ethiopia. *Environmental Science & Policy*, *90*, 173–182. <https://doi.org/10.1016/j.envsci.2018.01.018>

Subedi, R., Karki, M., & Panday, D. (2020). Food system and water–energy–biodiversity nexus in Nepal: A review. *Agronomy*, *10*(8), 1129.

Sušnik, J., Masia, S., Indriksone, D., Brēmere, I., & Vamvakeridou-Lydroudia, L. (2021). System dynamics modelling to explore the impacts of policies on the water-energy-food-land-climate nexus in Latvia. *Science of The Total Environment*, *775*, 145827.

Takaes Santos, I. (2020). Confronting governance challenges of the resource nexus through reflexivity: A cross-case comparison of biofuels policies in Germany and Brazil. *Energy Research & Social Science*, *65*, 101464. <https://doi.org/10.1016/j.erss.2020.101464>

Tesfaye, M., Moges, S., Melesse, A., & Agide, Z. (2021). Long-term water–energy–food security and resources sustainability: A case study of Ethiopia by 2030 and 2050. *International Journal of Energy and Water Resources*, 1–14.

Tyagi, N. K. (2020). Managing Water–Energy–Food Security Nexus Under Changing Climate: Implementation Challenges and Opportunities in India. *Transactions of the Indian National Academy of Engineering*, *5*(3), 449–464.

Tyagi, N., & Mehta, L. (2017). *Understanding water–energy–food security nexus to design technology and policy approaches for enhanced adaptation to climate change in India: Report of researcher exchange June 2017*.

Urbinatti, A. M. (2020). *Nexos de sustentabilidade e intersetorialidade: Políticas públicas em periferias urbanas*.

Urbinatti, A. M., Benites-Lazaro, L. L., Carvalho, C. M. de, & Giatti, L. L. (2020). The conceptual basis of water-energy-food nexus governance: Systematic literature review using network and discourse analysis. *Journal of Integrative Environmental Sciences*, *17*(2), 21–43.

Vats, G. (2019). *A nexus approach to energy, water, and food security policy making in India*.

Venghaus, S., Märker, C., Dieken, S., & Siekmann, F. (2019). Linking environmental policy integration and the water-energy-land-(food-) nexus: A review of the European Union’s energy, water, and agricultural policies. *Energies*, *12*(23), 4446.

Vinca, A., Parkinson, S., Byers, E., Burek, P., Khan, Z., Krey, V., Diuana, F. A., Wang, Y. P., Ilyas, A., Koberle, A. C., Staffell, I., Pfenninger, S., Muhammad, A., Rowe, A., Schaeffer, R., Rao, N. D., Wada, Y., Djilali, N., & Riahi, K. (2020). The NExus Solutions Tool (NEST) v1.0: An open platform for optimizing multi-scale energy-water-land system transformations. *Geoscientific Model Development*, *13*(3), 1095–1121. <https://doi.org/10.5194/gmd-13-1095-2020>

Vinca, Adriano, Parkinson, S., Byers, E., Burek, P., Khan, Z., Krey, V., Diuana, F. A., Wang, Y., Ilyas, A., Köberle, A. C., Staffell, I., Pfenninger, S., Muhammad, A., Rowe, A., Schaeffer, R., Rao, N. D., Wada, Y., Djilali, N., & Riahi, K. (2020). The NExus Solutions Tool (NEST) v1.0: An open platform for optimizing multi-scale energy–water–land system transformations. *Geoscientific Model Development*, *13*(3), 1095–1121. a9h.

Vinca, Adriano, Parkinson, S., Willaarts, B., Magnuszewski, P., Byers, E., Burek, P., Ilyas, A., Wang, Y., Bhattacharya, A., Yogeswaran, N., Siddiqi, A., Thambi, S., Khan, A., Wada, Y., Muhammad, A., Krey, V., Djilali, N., & Riahi, K. (2019). Achieving Climate-Land-Energy-Water Sustainable Development Goals in the Indus Basin. *Geophysical Research Abstracts*, *21*, 1–1. a9h.

Xia, C., & Chen, B. (2020). Urban land-carbon nexus based on ecological network analysis. *Applied Energy*, *276*, 115465. <https://doi.org/10.1016/j.apenergy.2020.115465>

Xu, Z., Chen, X., Liu, J., Zhang, Y., Chau, S., Bhattarai, N., Wang, Y., Li, Y., Connor, T., & Li, Y. (2020). Impacts of irrigated agriculture on food–energy–water–CO 2 nexus across metacoupled systems. *Nature Communications*, *11*(1), 1–12.

Zhang, P., Zhang, L., Chang, Y., Xu, M., Hao, Y., Liang, S., Liu, G., Yang, Z., & Wang, C. (2019). Food-energy-water (FEW) nexus for urban sustainability: A comprehensive review. *Resources, Conservation and Recycling*, *142*, 215–224.

Zisopoulou, K., Karalis, S., Koulouri, M.-E., Pouliasis, G., Korres, E., Karousis, A., Triantafilopoulou, E., & Panagoulia, D. (2018). Recasting of the WEF Nexus as an actor with a new economic platform and management model. *Energy Policy*, *119*, 123–139.

Zizopoulos, G. (2017). *Water-Energy Nexus, Putting Pieces Together: Critical Review And Perspectives*.
