# Supplementary material for: Exploring Cross-Sectoral Implications of the Sustainable Development Goals: Towards a Framework for Integrating Health Equity Perspectives With the Land-Water-Energy Nexus
Source: Public Health Rev. 2022 May 11;43:1604362. doi: 10.3389/phrs.2022.1604362 (PMC9131490; doi:10.3389/phrs.2022.1604362)

**SEARCH TERMS USED ACROSS DATABASES.**

1. **Science Direct Search Terms**

"resource nexus" OR (land,water, energy) AND sdgs AND (cross-sectoral OR integrative OR co-production) AND "watershed" AND ("health equity" OR "socio-ecological determinants of health")

1. **Google Scholar**

(“sdgs OR sustainable development goals”) AND ("integrative OR cross-sectoral”) AND ~("watershed" OR "transboundary") AND ("resource nexus" OR "land, water, energy" OR "food, water, energy) AND (“eco-social OR health OR equity)

**To add Indigenous concept on Google Scholar**

(“sdgs OR sustainable development goals”) AND ("integrative OR cross-sectoral”) AND ~("watershed" OR "transboundary") AND ("resource nexus" OR "land, water, energy" OR "food, water, energy) AND (“eco-social OR health OR equity) AND indigenous OR decolonizing OR ecological knowledge)

1. **Search terms for Web of Science, Academic Search Complete and Medline OVID**

| **Watershed** | (watershed* OR catchment* OR basin* OR river* OR river basin OR river-basin OR wetland* OR transboundary water OR delta) |
| --- | --- |
| **SDGs** | ("sustainable development goals" OR "sustainable development agenda" OR "sustainability goals" OR "global goals" OR sdg* OR "un agenda" OR "2030 agenda") |
| **Resource Nexus** | (("resource nexus" OR "resource synergies" OR "resource integration") OR (land, water, energy) OR (land-water-energy) OR (water, food, energy) OR (water-food-energy) OR (food, water, land, energy) OR (food-water-land-energy)) |
| **Cross-sectoral** | (cross-sectoral OR *sectoral OR integrat* OR *linkages OR *stakeholder OR co$production OR synergies OR collaborati*) |
| **Health Equity** | ((("health equity" OR "health disparit*" OR "social determinants of health") OR ("health of indigenous people" OR "women* health" OR "low income communities" OR "rural communities" OR "community health" OR "socio$economic status" OR "marginalized group$" OR vulnerab*) OR ("distributive justice" OR "water justice" OR "social justice" OR "environmental justice" OR co$benefits OR trade-off*) OR (*equity OR *equality OR "human well$being" OR livelihoods OR gender OR "human rights" OR intersectionality) OR ("power sharing" OR exclusion OR inclusion OR minorit* OR discriminat* OR "social status" OR marginaliz* OR "gender equality" OR representation OR access) OR ("food security" OR "fish extinction" OR "tree mortality" OR wildlife OR "stream contamination" OR "groundwater pollution" OR "forest cover" OR "habitat protection" OR "biodiversity loss and impacts" OR "* extinction" OR "environmental impacts" OR "eco$social impacts" OR ​"biodiversity conservation" OR  ("* ecological knowledge"​ OR "decolonizing health"))) |
| **Indigeneity** | (indigen* OR decoloniz* OR aboriginal OR "* ecological knowledge" OR "first nations" OR metis OR inuit OR "native people") |

1. **Web of Science Returned Searches**


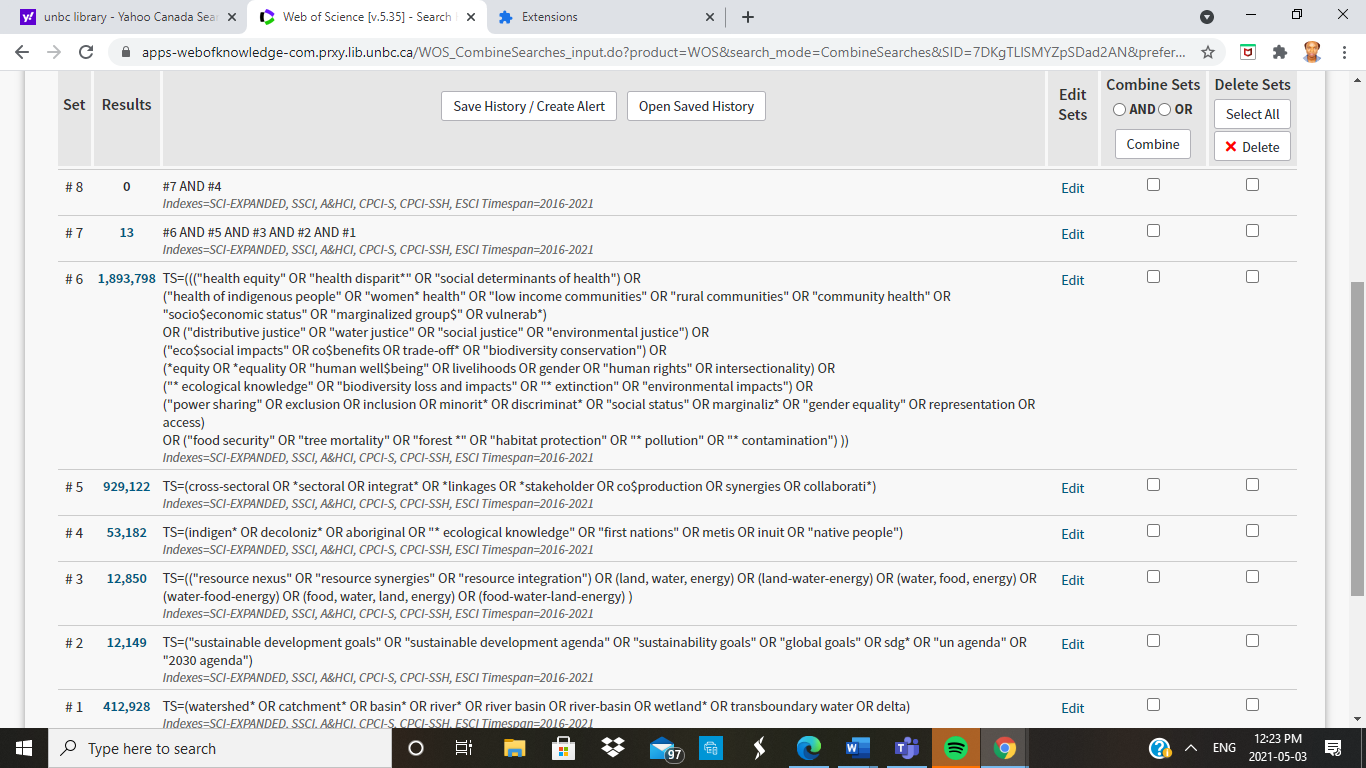


1. **An Excerpt of the Literature found in Web of Science.**


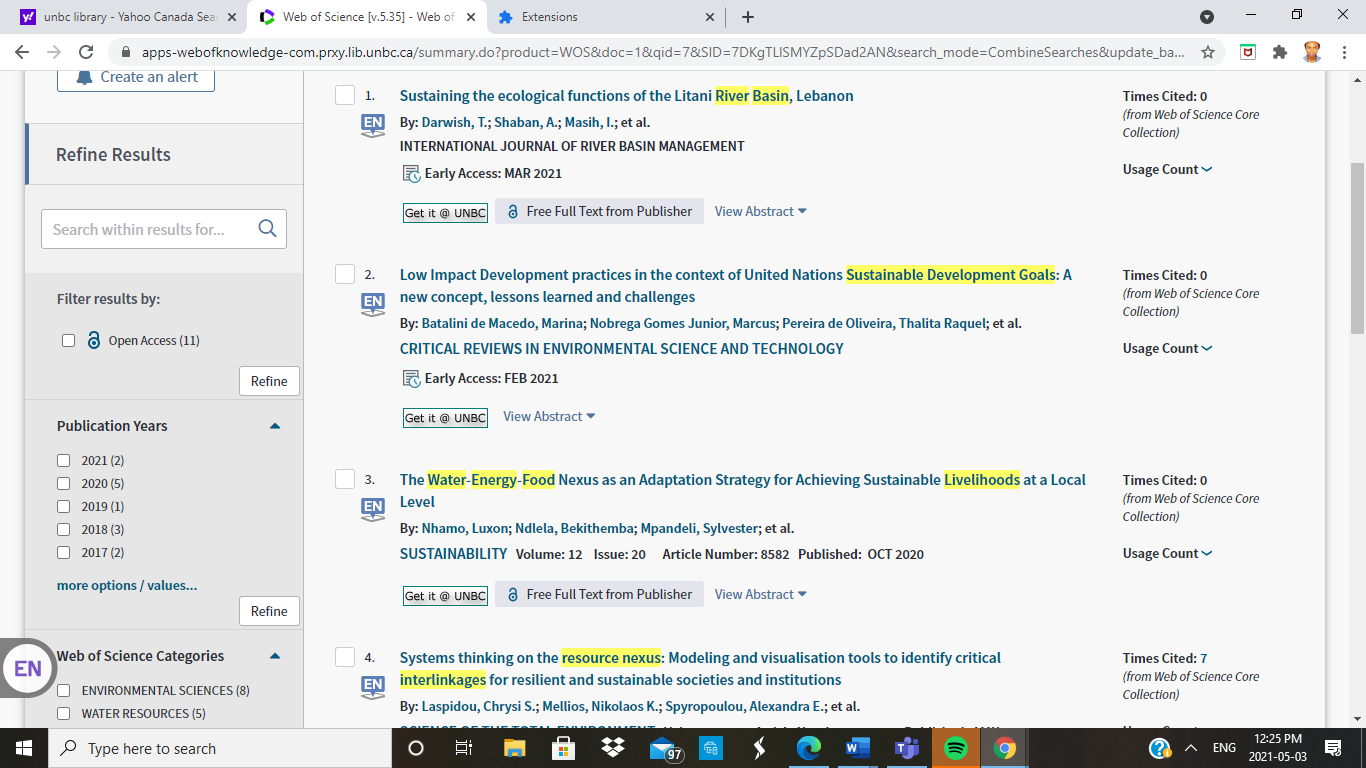


1. **Academic Search Complete Returned Searches**


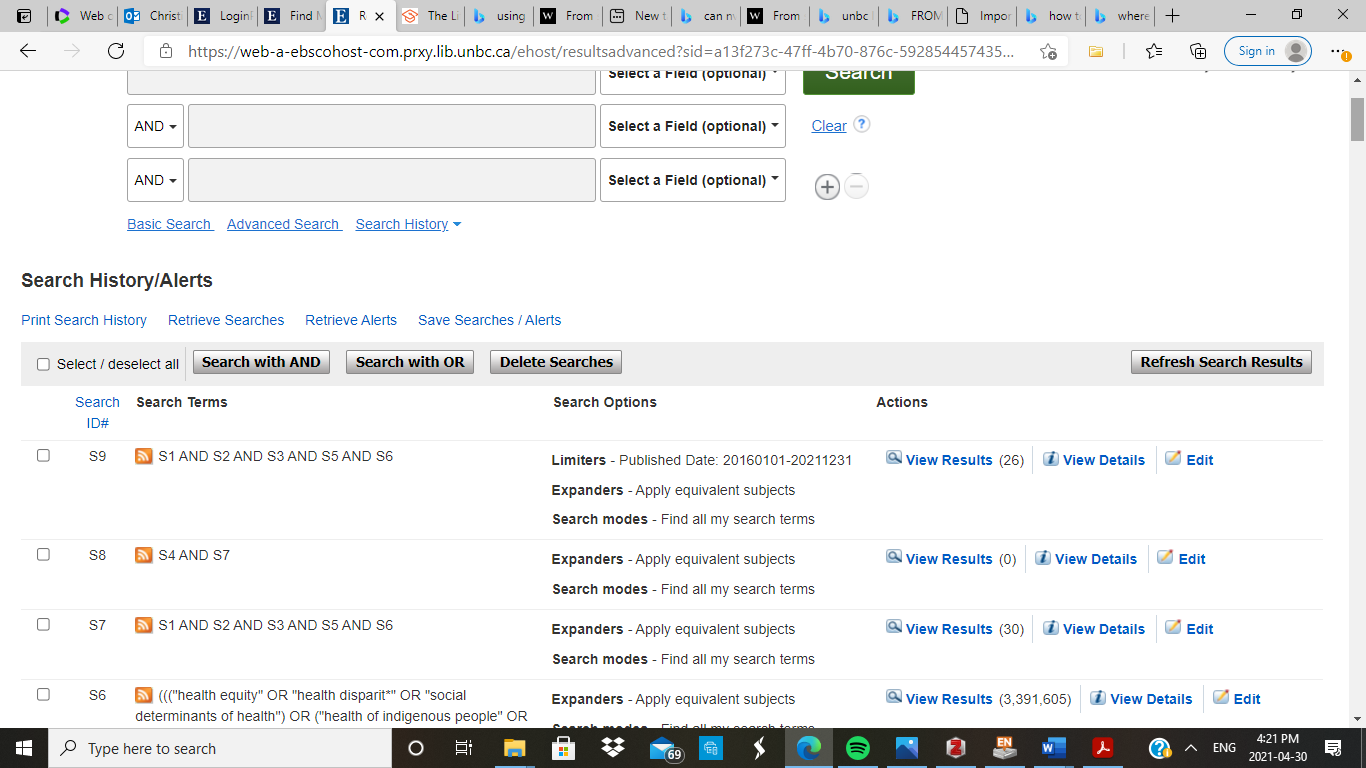


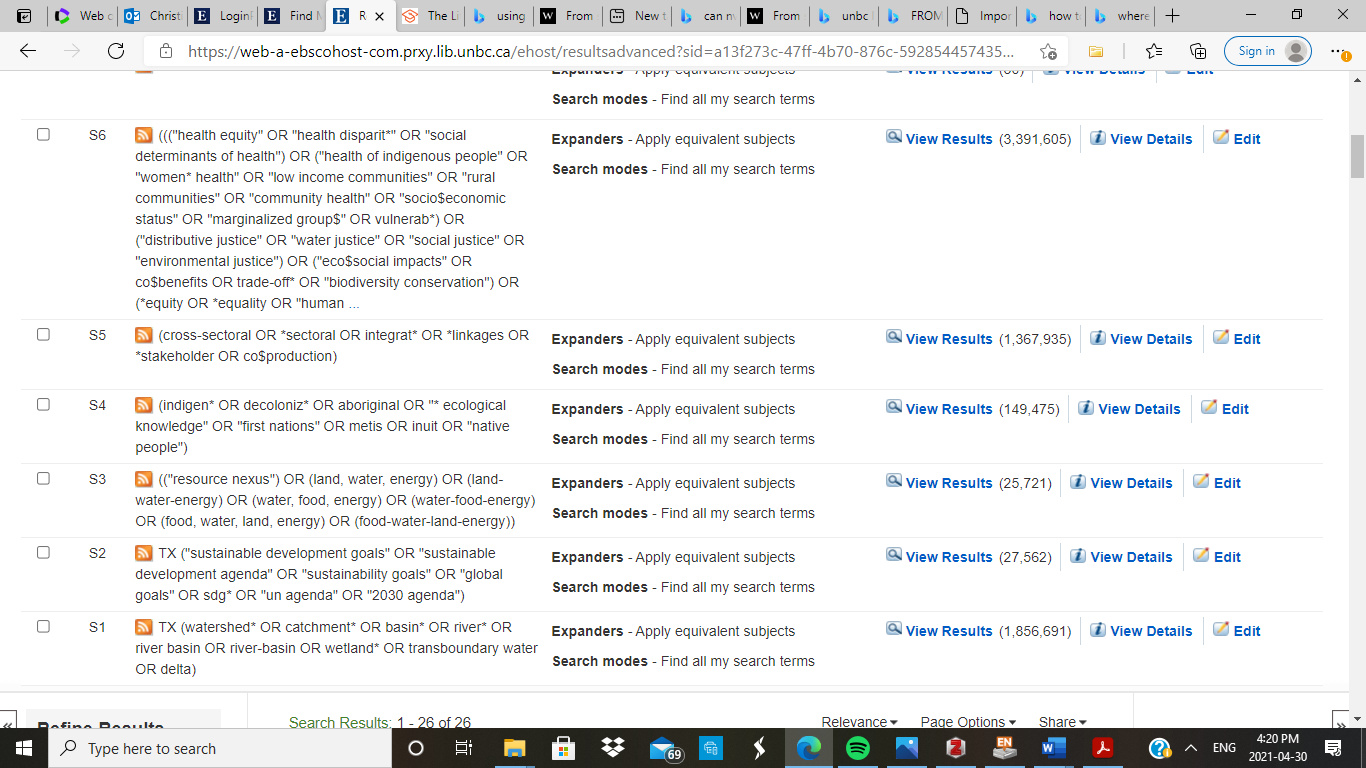


1. **An Excerpt of the Literature found in Academic Search Complete.**


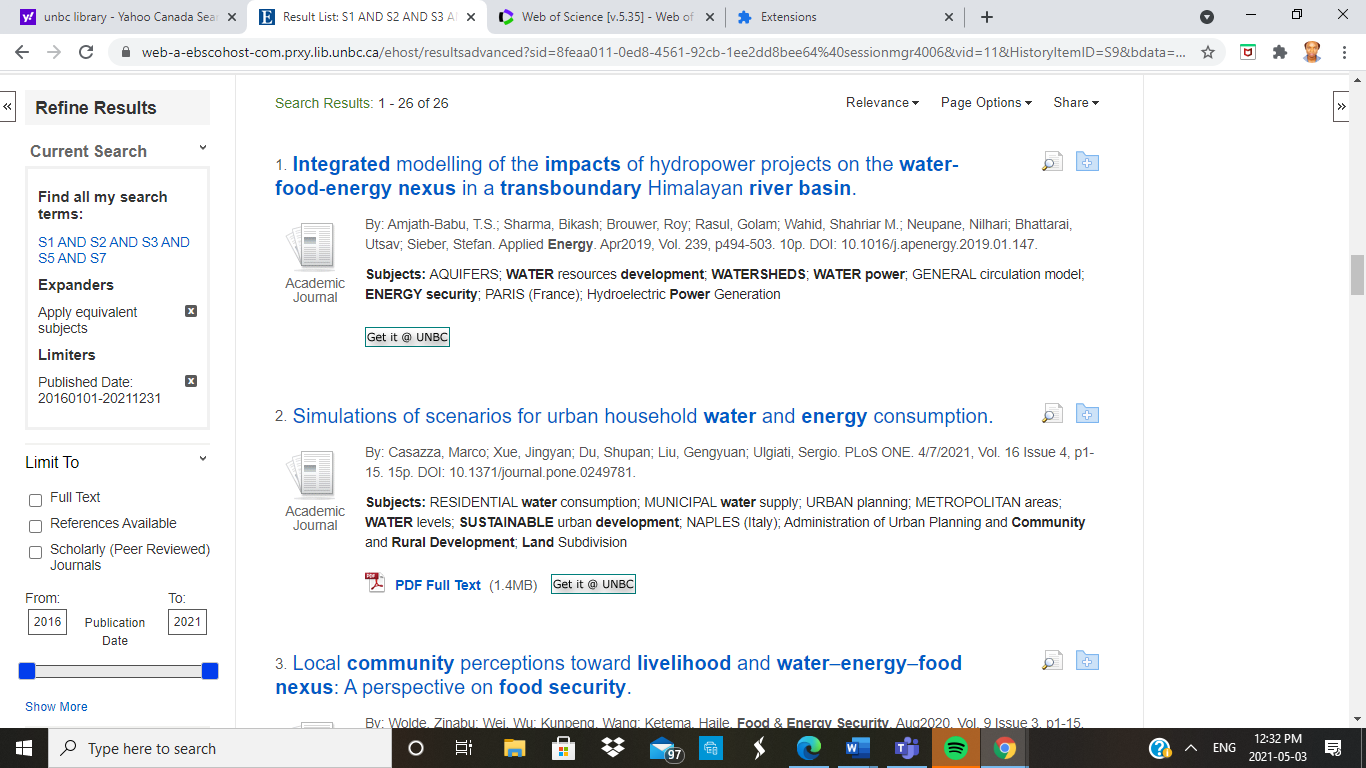


1. **Science Direct Returned Searches**


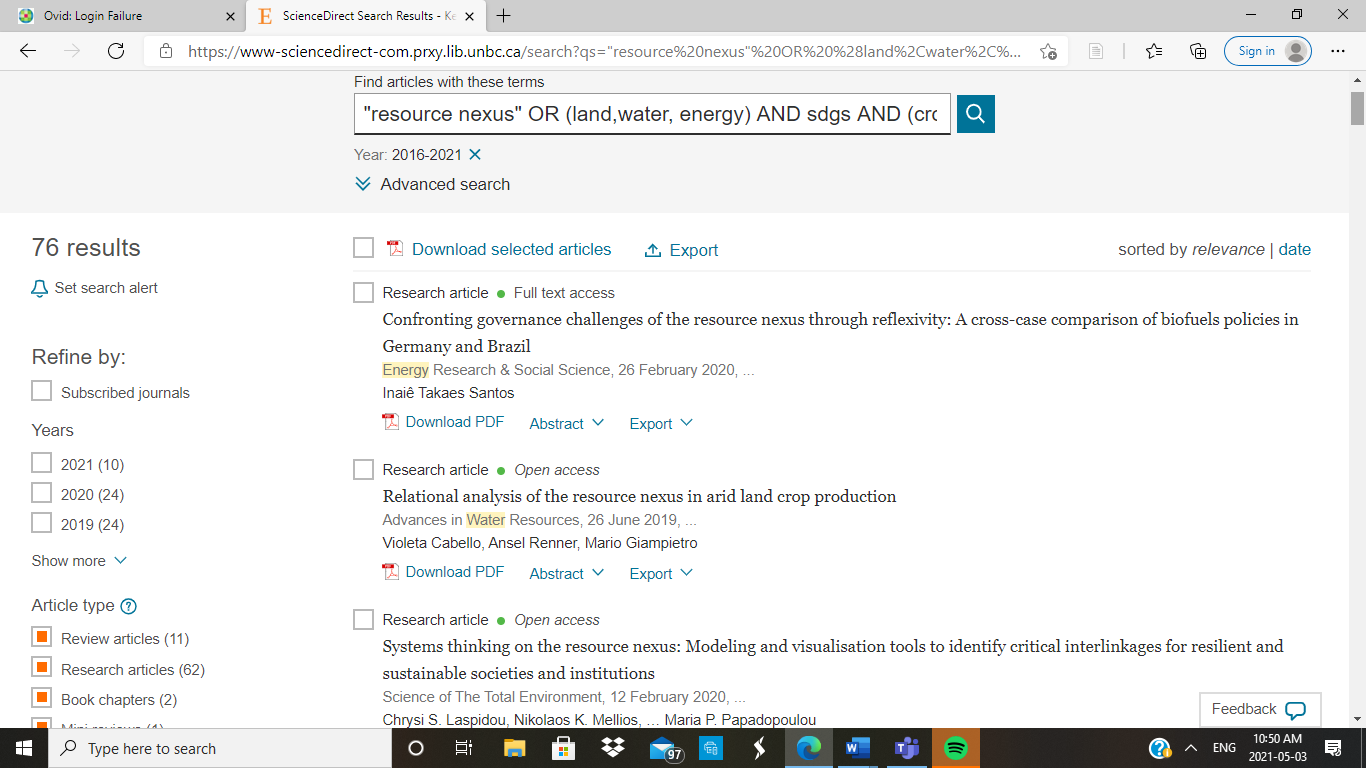


1. **Google Scholar Returned Searches**


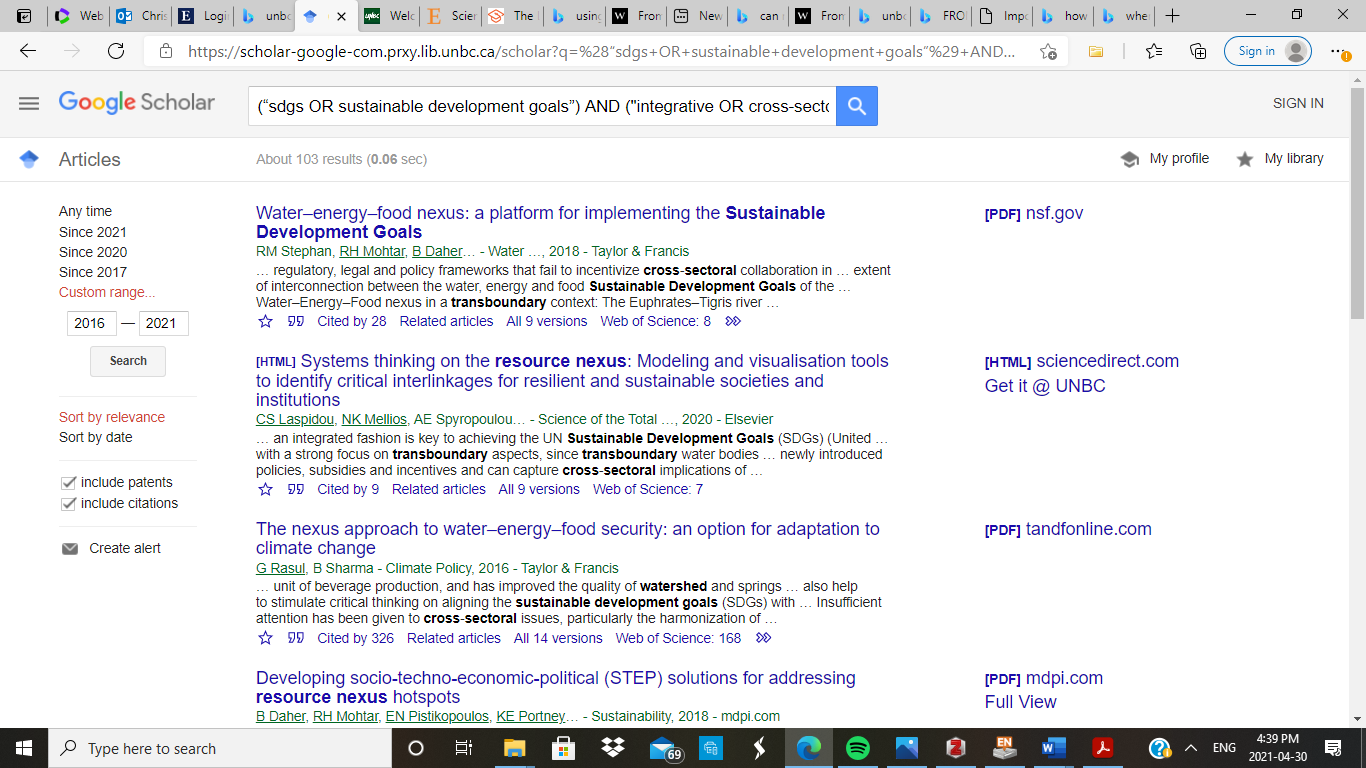

Supplement: Supplementary file 4 [file Table3.DOCX]
