# Supplementary material for: Exploring Cross-Sectoral Implications of the Sustainable Development Goals: Towards a Framework for Integrating Health Equity Perspectives With the Land-Water-Energy Nexus
Source: Public Health Rev. 2022 May 11;43:1604362. doi: 10.3389/phrs.2022.1604362 (PMC9131490; doi:10.3389/phrs.2022.1604362)
Supplement: Supplementary file 5 [file Table4.DOCX]

**PICO-Adapted Framework Used to Guide Search and Data Extraction**

| **PICO- (Adapted Framework** | **Thematic Information collated** |
| --- | --- |
| Problem/Perspective | Research problem, question, research perspectives or aim articulated |
| Intervention | The SDGs, as in the context of this research is the intervention. Or any conceptual or theoretically underpinnings of cross-sectoral considerations |
|  | Settings: urban, rural transboundary (as in watershed, river basin |
| Outcome | Integration, cross-sectoral possibilities |
| Evaluation | Limitations/Gaps/Opportunities |
|  |  |

***Inclusion Criteria for Screening***

**Article was included if it satisfied at least one of the *first three* conditions in the inclusion criteria.**

***Discusses the SDGs and cross-linkages through the lens of a nexus approach**

***Focuses on interplay of actors or sectors or interest and distribution of power, resources and impacts in a watershed context or in the context of land/food, water, energy nexus.**

***Engages with integrative, cross-sectoral, or indigenous perspectives in a watershed context or in the context of land/food, water, energy nexus**

- **Includes articles from anywhere in the world**
- **Includes only studies from 2016-2021**
- **Includes peer-reviewed articles, conference publications and grey literature.**
- **Published in English.**
